# RANBP2 Activates *O*-GlcNAcylation through Inducing CEBPα-Dependent OGA Downregulation to Promote Hepatocellular Carcinoma Malignant Phenotypes

**DOI:** 10.3390/cancers13143475

**Published:** 2021-07-12

**Authors:** Xiaoming Liu, Xingyu Chen, Mengqing Xiao, Yuxing Zhu, Renjie Gong, Jianye Liu, Qinghai Zeng, Canxia Xu, Xiong Chen, Fen Wang, Ke Cao

**Affiliations:** 1 Department of Oncology, Third Xiangya Hospital of Central South University, Changsha 410013, China; liuxiaoming26@csu.edu.cn (X.L.); xingyuchen@csu.edu.cn (X.C.); xiaomengqing@csu.edu.cn (M.X.); zhuyxx@csu.edu.cn (Y.Z.); 2 Department of Gastroenterology, Third Xiangya Hospital of Central South University, Changsha 410013, China; 13028686810@163.com (R.G.); xucanxia@csu.edu.cn (C.X.); chenxiong-1225@163.com (X.C.); wfen-judy@csu.edu.cn (F.W.); 3 Department of Urology, Third Xiangya Hospital of Central South University, Changsha 410013, China; ljyxy3yy@csu.edu.cn; 4 Department of Dermatology, Third Xiangya Hospital of Central South University, Changsha 410013, China; zengqinghai@csu.edu.cn

**Keywords:** RANBP2, SUMOylation, *O*-GlcNAcylation, CEBPα, OGA, hepatocellular carcinoma

## Abstract

**Simple Summary:**

Hepatocellular carcinoma (HCC) is characterized by a poor prognosis and high mortality rate, with complex molecular alterations, including glycosylation. O-linked N-acetylglucosamine (O-GlcNAc) is a dynamic modification process jointly controlled by the “writer” *O*-GlcNAc transferase (OGT) and “eraser” *O*-GlcNAcase (OGA), and an increase in total *O*-GlcNAc correlates with advanced malignant HCC phenotypes. The aim of our study was to explore the potential regulatory patterns underlying the OGT/OGA imbalance that contributes to HCC malignancies. We confirmed that RANBP2, one of the SUMO E3 ligases, downregulates OGA transcription while not affecting OGT. As a transcriptional factor positively regulating OGA, CEBPα was also SUMOylated and destabilized by RANBP2. Our in vitro and in vivo studies provide evidence of RANBP2 downregulating OGA and thus triggering *O*-GlcNAcylation in a CEBPα-dependent manner. The subsequent hyper-*O*-GlcNAcylation of protein substrates such as PGC1α via the RANBP2–CEBPα–OGA pathway may represent a pharmaceutical strategy for HCC treatment.

**Abstract:**

*O*-GlcNAcylation is an important post-translational modification (PTM) jointly controlled by *O*-GlcNAc transferase (OGT) and *O*-GlcNAcase (OGA). Aberrant hyper-*O*-GlcNAcylation is reported to yield hepatocellular carcinoma (HCC) malignancy, but the underlying mechanisms of the OGT/OGA imbalance responsible for HCC tumorigenesis remain largely unknown. Here, we report that RAN-binding protein 2 (RANBP2), one of the small ubiquitin-like modifier (SUMO) E3 ligases, contributed to malignant phenotypes in HCC. RANBP2 was found to facilitate CCAAT/enhancer-binding protein alpha (CEBPα) SUMOylation and degradation by direct interplay with CEBPα. As a transcriptional factor, CEBPα was verified to augment OGA transcription, and further experiments demonstrated that RANBP2 enhanced the *O*-GlcNAc level by downregulating OGA transcription while not affecting OGT expression. Importantly, we provided in vitro and in vivo evidence of HCC malignant phenotypes that RANBP2 triggered through an imbalance of OGT/OGA and subsequent higher *O*-GlcNAcylation events for oncogenic proteins such as peroxisome proliferative-activated receptor gamma coactivator 1 alpha (PGC1α) in a CEBPα-dependent manner. Altogether, our results show a novel molecular mechanism whereby RANBP2 regulates its function through CEBPα-dependent OGA downregulation to induce a global change in the hyper-*O*-GlcNAcylation of genes, such as PGC1α, encouraging the further study of promising implications for HCC therapy.

## 1. Introduction

Liver cancer has become one of the most common malignancies in China and the third most lethal neoplasm worldwide [1]. Hepatocellular carcinoma (HCC), accounting for more than 80% of all liver cancer cases, has a poor prognosis and high mortality rate worldwide [2]. It has been widely accepted that the occurrence, progression, and prognosis of HCC may be accompanied by complex molecular alterations, including genetic and epigenetic changes. Current investigations are focused on better understanding the disease-relevant molecular mechanisms, which is indispensable for novel targeted therapy; post-translational modifications (PTMs) such as glycosylation are emerging for expanding the therapeutic repertoire [3,4].

*O*-linked β-N-acetylglucosaminylation (*O*-GlcNAcylation) is a highly prevalent and dynamic PTM in multicellular organism studies. It is a unique type of glycosylation whereby a single sugar moiety, *O*-linked N-acetylglucosamine (*O*-GlcNAc), is typically transferred to the hydroxyl groups of serine and threonine residues of proteins [5]. Although hypothesized to exist, *O*-GlcNAc’s cycling is only known to be regulated by one “writer” for protein *O*-GlcNAcylation, namely, *O*-GlcNAc transferase (OGT), and one “eraser”, called *O*-GlcNAcase (OGA) [6]. *O*-GlcNAc homeostasis plays important roles in cell signaling and gene regulation [7], but how this regulation is fine-tuned is not well understood. It was recently reported that disrupted *O*-GlcNAc is observed in pancreatic cancer [8]. Studies on HCC phenotypes detected elevated *O*-GlcNAc levels for certain proteins that profoundly affect hepatocarcinogenesis, such as PGC1α (peroxisome proliferative-activated receptor gamma coactivator 1 alpha) [9], c-MYC [10], and RACK1 (ribosomal receptor for activated C-kinase 1) [11]. Abnormal *O*-GlcNAc glycosylation is found in various tumors, and specifically, an increase in total *O*-GlcNAc has been detected in HCC compared to normal tissue [12].

As *O*-GlcNAc-cycling enzymes, both the writer and eraser act to regulate the presence of *O*-GlcNAcylation; changes in this dynamic for over a thousand protein substrates are therefore almost entirely dependent on the OGT/OGA balance. The above phenomena raise an interesting question of how the accumulating *O*-GlcNAcylation triggers HCC tumorigenesis. It was recently reported that UAP1 and UAP1L1 cooperatively activate OGT to promote *O*-GlcNAcylation [10]; however, the exact mechanisms governing OGT/OGA imbalance remain obscure. The upstream molecules for OGA deregulation may provide novel insights into the biological process of HCC malignant transformation, yet this accurate control has not been reported in all types of cancer.

SUMOylation is a multi-step reaction sequentially catalyzed by a SUMO-activating E1 enzyme, the single conjugating E2 enzyme Ubc9, and E3 ligase. The whole process is analogous to the cascade reaction of ubiquitination [13]. The RAN-binding protein 2 (RANBP2), a small ubiquitin-like modifier (SUMO) E3 ligase, is a 358-kDa nucleoporin that participates in the nuclear pore complex (NPC) [14]. RANBP2 has multiple domains, which can be utilized as potential “on/off” nuclear/cytoplasm switches by various proteins such as exportin-1/CRM1 [15], cox11 [16], and importin-β [17]. Evidence shows that SUMOylation and cyto-nuclear shuttling by RANBP2 are tightly connected, as it is the major component of NPC cytoplasmic filaments on the nuclear membrane [18]. RANBP2 was recently found to be significantly upregulated in HCC tumorigenesis by our group, but how RANBP2 behaves in HCC is not clear [19]. The data in the current study identify RANBP2 as a crucial biomarker correlating with HCC malignancy. The underlying mechanism of RANBP2′s action, involving the SUMOylation and subsequent downregulation of CCAAT/enhancer-binding protein alpha (CEBPα) by RANBP2, accounts for OGA transcriptional inhibition, which causes hyper-*O*-GlcNAcylation and promotes HCC progression.

## 2. Results

### 2.1. RANBP2 Is Enriched in HCC and Correlates with Malignant Phenotypes

**Figure 1:**
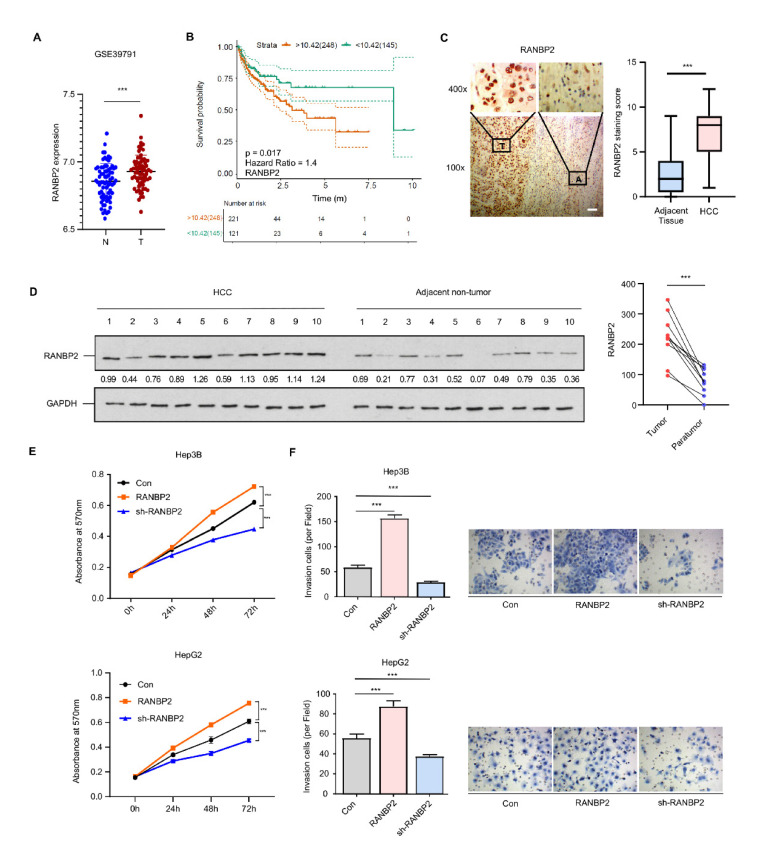
RANBP2 is enriched in HCC and correlates with malignant phenotypes. (**A**) RANBP2 mRNA level comparison between HCC and adjacent non-tumor tissue from the GEO databases. *** *p* < 0.001, *t*-test. (**B**) Survival curve from the GEPIA database indicates that RANBP2 was significantly associated with lower survival rate in HCC patients. The expressional tendency and clinical prognosis of RANBP2 in HCC are further shown in Supplementary Figure S1. (**C**) Immunohistochemical staining of RANBP2 in HCC tissue and matching adjacent non-tumor tissue. Left panel: Representative images (from total of 54 clinical samples) encompassing the border of tumor and non-tumor tissues. Right panel: Quantification of RANBP2 staining scores in box-and-whisker plot (plot: min to max). T = tumor tissue; A = adjacent non-neoplastic liver tissue. Scale bars, 20 µm. *** *p* < 0.001, χ^2^ test. (**D**) Western blotting of RANBP2 in HCC tissue and matching non-neoplastic tissue. The paired gray values are shown in the right panel; n = 10 patients. (**E**,**F**) Cell viability (**E**) and invasion (**F**) in response to altered RANBP2. CCK-8 assay was conducted after 24, 48, and 72 h. The invasion ability was determined by Transwell assay. Hep3B and HepG2 cells were transfected with a RANBP2-overexpressing vector and targeting shRNAs. Data are presented as the mean ± SD values from three biological replicates. *** *p* < 0.001, *t*-test. RANBP2 knockdown efficiency is shown in Supplementary Figure S2A.

Analysis of multiple datasets from the Gene Expression Omnibus (GSE39791, GSE33006, and GSE46408) revealed RANBP2 to be significantly enriched in HCC versus normal tissue (Figure 1A and Figure S1A). RANBP2 was also associated with a lower overall survival rate in HCC patients from both public data (Figure 1B) and our clinical samples (50 cases having follow-up information among total 54 patients; see Supplementary Figure S1B). This was in accordance with our previous study showing that an increase in RANBP2 induced by the deacetylase SIRT1 contributed to HCC tumorigenesis [19]. In the current study, RANBP2 expression was detected in the liver tissue of HCC patients, and the correlation of RANBP2 levels with patients’ survival rates was analyzed. According to the immunohistochemistry (Figure 1C) and Western blot results (Figure 1D), HCC tissues had more RANBP2 than adjacent non-tumor ones. In our HCC tissues (*n* = 54), RANBP2 levels were highly correlated with neoplastic grade (Edmondson–Steiner, *p* = 0.016), pT status (*p* = 0.042), and tumor size (*p* < 0.001) (Supplementary Table S1). According to the results of CCK-8 and Transwell assays, RANBP2 overexpression caused increasing proliferation in both Hep3B and HepG2 cells, while its deletion had opposite effects (Figure 1E,F; the knockdown efficiencies of RANBP2 were shown in Supplementary Figure S2A). These data collectively indicate that a high level of RANBP2 correlates with HCC malignant phenotypes.

### 2.2. Delineation of RANBP2 Functions via Interaction with CEBPα and Negatively Regulating Its Expression

**Figure 2:**
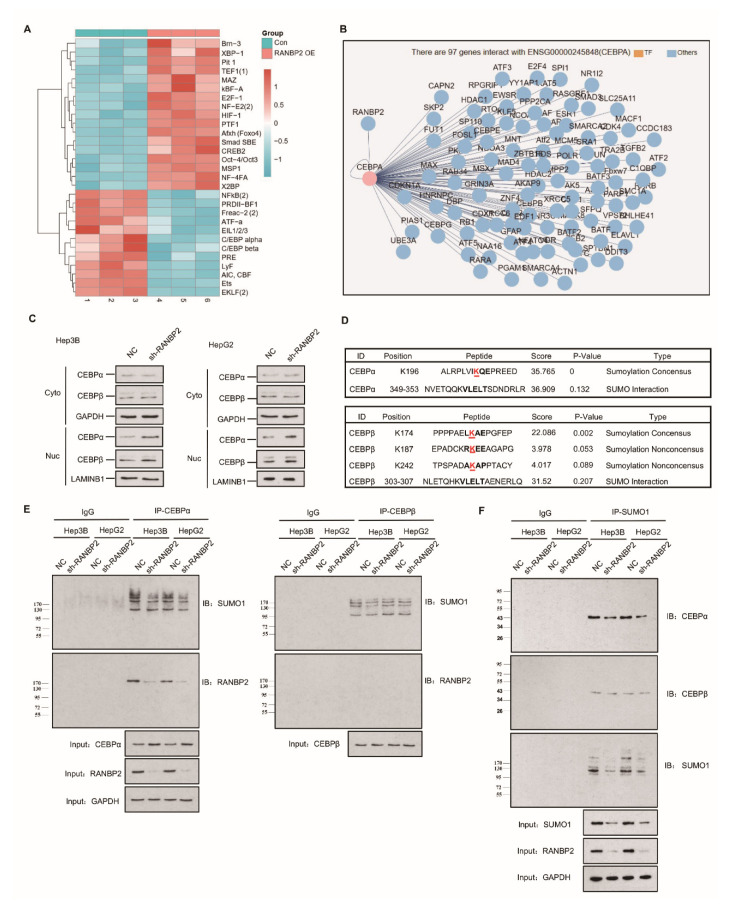
Endogenous RANBP2 interacts with CEBPα and downregulates its expression in HCC cells. (**A**) The transcriptional factor array revealed the potential downstream targets of RANBP2. The CEBP family, including CEBPα and CEBPβ, were significantly downregulated in RANBP2-overexpressing Hep3B cells. (**B**) The potential interplay between RANBP2 and the transcriptional factor CEBPα. Though a crucial member of the same protein family, CEBPβ was not predicted to interact with RANBP2. (**C**) Cell fractionation assays showed increased CEBPα and CEBPβ nuclear expression but no cytoplasmic alteration upon RANBP2 knockdown. Two independent experiments were conducted. GAPDH and LAMINB1 were used as controls in cytosol and nuclei, respectively. The additional immunofluorescence study, shown in Supplementary Figure S3, revealed that CEBPα and CEBPβ were inversely correlated with RANBP2. (**D**) Schematic representation of the predicted SUMO-conjugation motifs in the CEBPα and CEBPβ proteins. GPS-SUMO software indicated the predicted Lysine(K)-196 and Lysine(K)-174 SUMO-conjugation motifs and corresponding scores. (**E**) Co-immunoprecipitation confirmed the endogenous interaction of CEBPα and RANBP2. Additionally, immunoblotting with SUMO1 provided information of all RANBP2-associated SUMOylated proteins that potentially interacted with CEBPα. Meanwhile, no CEBPβ–RANBP2 binding was detected. Equal CEBPα or CEBPβ loading in the IP panel was pre-verified. (**F**) CEBPα served as a direct SUMO substrate of RANBP2. When immunoprecipitating with SUMO1 antibody, lower SUMOylated CEBPα and SUMO1 levels were detected in the RANBP2-depleted condition. By contrast, the predicted RANBP2 SUMO target of CEBPβ was not verified. Equal (pre-verified) SUMO1 was also loaded in the IP panel.

The high expression of RANBP2 in HCC encouraged us to examine its contribution and related regulatory mechanisms in HCC development. The concept of small ubiquitin-related modifier (SUMO) participating in cytosol–nucleus shuttling through direct protein–protein interaction or transient post-translational regulation is being actively investigated, but most of these nuclear–cytoplasmic movements are believed to be bidirectional [20]. Among various regulatory molecules, the dogma of transcriptional factor (TF) activity being characterized by a high nuclear–cytoplasmic distribution ratio (N/C) led us to identify a certain unidirectional nuclear influx regulated by RANBP2. Transcriptional factor arrays were then used to explore potential downstream targets of RANBP2. The CEBP family, including CEBPα and CEBPβ, gained high scores due to their significant downregulation upon RANBP2 overexpression (Figure 2A), similar to previous reports on the HCC-suppressive function of CEBPα [21], in contrast to the promoting role of CEBPβ [22]. These TFs with highly debatable involvement in HCC progression came to light as possibly further serving as intermediates of RANBP2 and HCC progression, precisely because RANBP2 is a nuclear protein normally excluded from the cytoplasm by the nuclear pore complex (NPC) [23]. When assessing the network of the RANBP2 protein, CEBPα was shown to interact with RANBP2 (Figure 2B). Cell fractionation assays and immunofluorescence studies confirmed that CEBPα was mainly retained in nuclei, and an increased nuclear abundance was detected in RANBP2-knockdown conditions. The faint signal for CEBPα in the cytosol predominantly revealed its acknowledged transcriptional property. Moreover, given the dynamic interaction between the family members CEBPα and CEBPβ during liver regeneration, CEBPβ’s distribution was also investigated to confirm the pertinent role of RANBP2 [24]. Though not predicting the potential RANBP2–CEBPβ interaction, stronger signals of CEBPβ were also detected in the absence of RANBP2 (Figure 2C and Figure S3). These results show the downregulation of both CEBPα and CEBPβ in the presence of RANBP2.

Apart from the interplay with TFs, SUMOylation may significantly underlie RANBP2′s oncogenic property. Bioinformatics analyses using the GPS-SUMO tool predicted one and three SUMO consensus motifs for CEBPα (K196) and CEBPβ (K174, K187, and K242), respectively, with high scores (Figure 2D). To assess the regulatory role of RANBP2 for both CEBPα and CEBPβ, we first carried out experiments on endogenous protein–protein interactions. Immunoblotting with an anti-RANBP2 antibody for immunoprecipitated CEBPα verified an RANBP2–CEBPα interaction, while CEBPβ was unable to precipitate RANBP2. In addition, when immunoblotting with an anti-SUMO1 antibody, all the SUMOylated proteins that potentially interacted with CEBPα were shown, and the fainter band in the RANBP2-depleted condition for two HCC cell lines revealed that RANBP2 affected SUMO proteins that interacted with CEBPα. By contrast, very faint and unchanged SUMO1 signals for CEBPβ were detected in control and RANBP2-depleted conditions. These results suggest a potential SUMOylating role of RANBP2 apart from physical interplay with CEBPα (Figure 2E). Next, for checking SUMO-CEBPα conjugates, lysates were immunoprecipitated with SUMO1 antibody. Lower SUMOylated CEBPα and total SUMO1 levels were found in the RANBP2-depleted condition, while the predicted RANBP2 SUMO target of CEBPβ was not verified (Figure 2F). These data indicate that RANBP2 downregulates CEBPα’s endogenous expression, possibly through protein–protein interaction and SUMOylation.

### 2.3. RANBP2 Overexpression Promotes CEBPα SUMOylation and Its Subsequent Degradation

**Figure 3:**
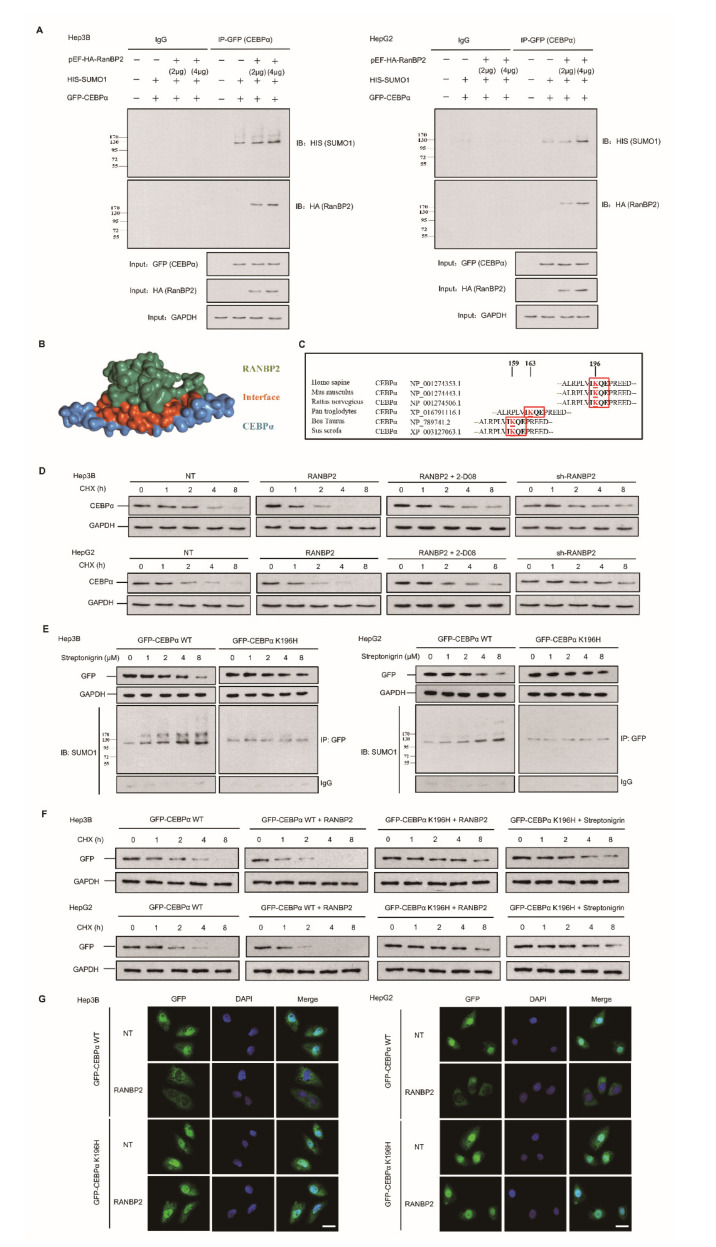
RANBP2-dependent SUMOylation of CEBPα promotes its instability in HCC cells. (**A**) RANBP2 exogenously co-precipitated CEBPα and increased SUMOylation of CEBPα in vitro. Hep3B and HepG2 cells were transfected with foreign pEF-HA-RanBP2 (2 and 4 μg in gradients), GFP-CEBPα, and HIS-SUMO1 plasmids. An increase in the SUMOylation of CEBPα was detected in RANBP2-overexpressing conditions. Additionally, the interaction of exogenous RANBP2 and CEBPα was confirmed. Equal GFP (CEBPα) loading in the IP panel was demonstrated, and the input of HA (RanBP2) and GAPDH is also shown (as a control). (**B**) The structural representation of RANBP2 and CEBPα interplay. The potential interacting and SUMOylation sites are highlighted as red sticks. (**C**) Comparison of amino acid sequences adjacent to K196 in CEBPα among the indicated vertebrate species. The evolutionarily conserved SUMOylation sites of CEBPα at lysine(K)-196 are depicted using red boxes for humans and another five species. (**D**) RANBP2 reduced endogenous CEBPα protein stability in HCC cells treated with cycloheximide (CHX, 10 mg/mL) at the indicated time points. Hep3B and HepG2 cells treated with RANBP2-overexpression plasmid showed lower CEBPα protein at indicated time points, while a relatively high expression of CEBPα was observed in the RANBP2-knockdown condition. Restoration of CEBPα protein was detected in RANBP2-overexpressing HCC cells treated with SUMO inhibitor 2-D08 (2′,3′,4′-trihydroxy flavone). (**E**,**F**) K196H blocked SUMOylation and slowed RANBP2-mediated CEBPα degradation. (**E**) WT CEBPα and K196H mutant were overexpressed in HCC cell lines, and then, cells were administered the SUMOylation agonist streptonigrin (SN) in a concentration gradient (0, 1, 2, 4, and 8 µM) for the indicated period (48 h). Exogenous CEBPα and SUMO1–CEBPα expression levels were detected by Western blotting and co-immunoprecipitation. Exogeneous WT CEBPα degradation with accumulating SUMOylation was found with increasing SN concentration. In the K196H mutant CEBPα group, SUMOylation was significantly reduced, and CEBPα remained relatively high in expression even in the condition of cumulative SN. (**F**) The CHX protein stability assay for exogenous CEBPα was performed under various conditions. HCC cells transfected with RANBP2-overexpressing vector showed faster WT CEBPα degradation, while prolonged K196H CEBPα expression was detected. Additionally, K196H CEBPα protein was used as a positive control and remained at a relatively high level even under treatment with the SUMO agonist streptonigrin (4 µM, the optimum concentration based on Figure 3E) at different time points compared to the WT CEBPα group. (**G**) SUMOylation of CEBPα K196 by RANBP2 delayed its localization and expression in nuclei. Ectopically expressed CEBPα (both WT and K196H) mainly localized in the nucleus. Downregulated WT CEBPα expression in nucleus was detected under RANBP2-overexpressing condition, whilst the abundance of CEBPα K196H was maintained. Immunofluorescence study was conducted with anti-GFP (green). DNA was stained with DAPI (blue). Merge, GFP + DAPI. Scale bars, 20 µm.

The pattern of regulation and fate of CEBPα following RANBP2 overexpression were further explored. We next overexpressed both RANBP2 and CEBPα in two HCC cell lines. Co-immunoprecipitation of exogeneous RANBP2 (HA-tagged) and CEBPα (GFP-tagged) was detected, and RANBP2 triggered the SUMOylation of CEBPα on the basis of equal CEBPα loading in the IP panel (Figure 3A). These results supplementally demonstrate the interaction of foreign CEBPα–RANBP2, which is in accordance with the structure diagram (shown in Figure 3B).

SUMOylation has long been known to affect the interactions, stability, localization, and activity of targeted proteins, but it is difficult to predict the consequences of SUMOylation at a specific site. Since the lysine-196 (K196) residue in CEBPα was identified as a SUMO site and was highly conserved among orthologues (Figure 3C), a protein stability assay was performed. Endogenous CEBPα protein expression was inhibited upon treatment with RANBP2 at an indicated time and was restored by the SUMO inhibitor 2-D08 (2′,3′,4′-trihydroxy flavone) group (Figure 3D). Next, a K196H mutation was utilized to confirm the site specificity of this SUMO effect. Under treatment with the SUMOylation agonist streptonigrin (SN) across a concentration gradient for 48 h, fewer SUMO–CEBPα and the restoration of CEBPα smears were observed in the SN-treated K196H group, indicating RANBP2-dependent CEBPα downregulation in the proteasomal pathway (Figure 3E). The stability of exogenous CEBPα was further investigated to determine the relevance of site-specific SUMOylation and protein fate. RANBP2 overexpression accelerated WT CEBPα degradation, while prolonged K196H CEBPα expression was detected. Additionally, the K196H CEBPα protein was used as a positive control and remained at a relatively high level even under treatment with the SUMO agonist SN at different time points compared to the WT CEBPα (Figure 3F). The immunofluorescence results indicated that both exogenous WT CEBPα and the K196H mutant mainly localized in the nucleus. Under RANBP2 overexpression, however, the downregulation of WT CEBPα in nuclei was detected, whilst the abundance of CEBPα K196H was unchanged (Figure 3G). These experiments with exogenous CEBPα confirmed that the SUMOylation of CEBPα K196 by RANBP2 delayed its localization and expression in nuclei. The above data further identify CEBPα as a direct SUMO target of RANBP2, and it is thus tempting to speculate that the SUMOylation of CEBPα contributes to its destabilization.

### 2.4. CEBPα Attenuates O-glycosylation through Triggering OGA Transcriptional Activity

**Figure 4:**
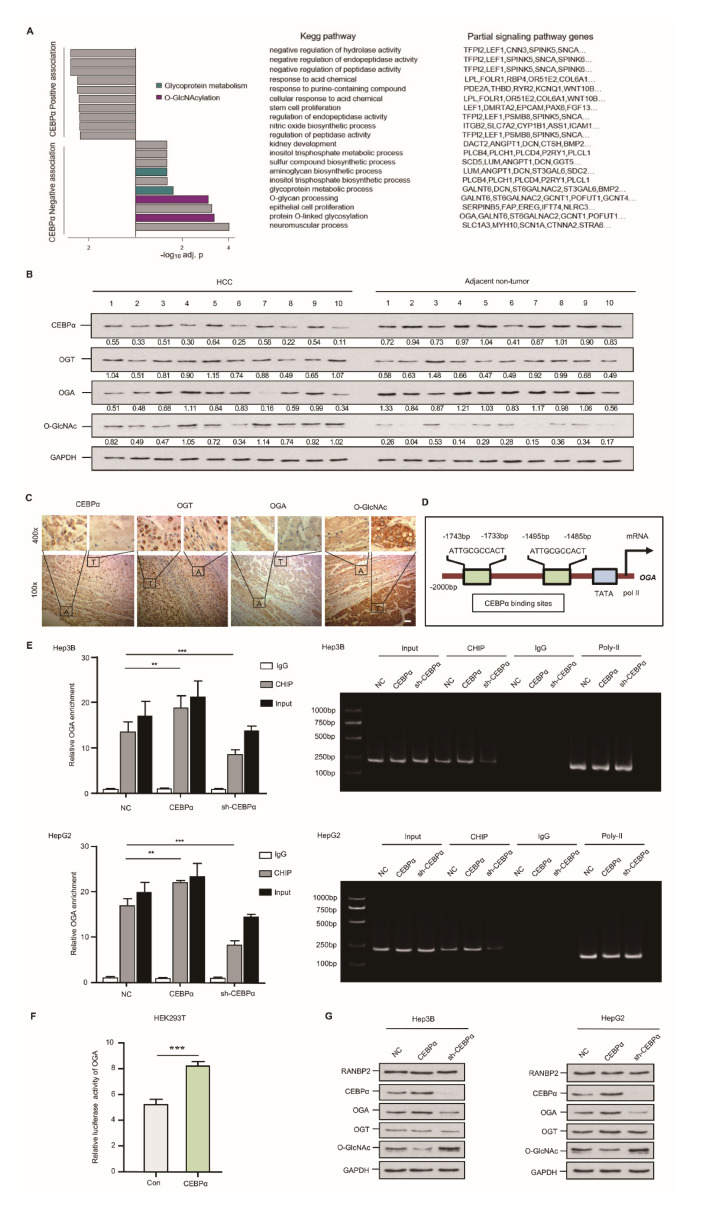
Downregulation of CEBPα is essential for OGA inactivation and hyper-*O*-GlcNAcylation in HCC. (**A**) CEBPα was negatively associated with response to *O*-GlcNAcylation and relevant glycoprotein metabolism. RNA assays followed by sh-CEBPα in Hep3B cells were conducted. *O*-GlcNAcylation and glycoprotein metabolism ranked top (4 out of 10 sets) in KEGG pathways; the typical signaling genes per set, such as OGA, are also shown. (**B**,**C**) Western blotting and immunohistochemical staining of CEBPα, OGT, OGA, and *O*-GlcNAc levels in HCC tissue and matching non-neoplastic tissue. The results indicate a positive correlation of CEBPα and OGA protein expression but a negative correlation of CEBPα protein and *O*-GlcNAc level. T = tumor tissue; A = adjacent non-neoplastic liver tissue. Scale bars, 20 µm. (**D**) Schematic binding prediction of transcriptional factor CEBPα in *OGA* gene promoter. The two positions and sequences are indicated. (**E**) Identification of CEBPα–OGA interaction using chromatin immunoprecipitation. Quantified expression (left panel) and representative agarose DNA electrophoresis (right panel) are displayed. CEBPα interferences were explored for both cell lines. CEBPα protein was able to pull down fragments of DNA encoding OGA. IgG and Input served as controls. The CEBPα knockdown efficiency was tested, as shown in Supplemental Figure S2B. (**F**) The binding of CEBPα in OGA promoter and its positive transcriptional regulation were confirmed by luciferase reporter assay. The ratio of firefly luciferase activity to Renilla activity was regarded as an index of the gene promoter activities. Each experiment was performed in triplicate. (**G**) Immunoblotting of *O*-GlcNAc and its homeostasis factors, including OGT and OGA. CEBPα resulted in significantly increased OGA, while OGT expression was unchanged, which was in accordance with the decrease in *O*-GlcNAc. RANBP2 was not affected by altered RANBP2 expression. Data are presented as the mean ± SD values from three biological replicates. ** *p* < 0.01, *** *p* < 0.001, *t*-test.

Using an RNA array and Hep3B cells, we identified the targets of CEBPα. CEBPα was silenced in a Hep3B cell line, with subsequent KEGG analysis (the knockdown efficiencies for CEBPα are shown in Supplementary Figure S2B). The top-scoring *O*-glycosylation and glycoprotein metabolism disorder known to favor HCC were negatively associated with CEBPα. Specifically, significantly downregulated OGA was observed in the CEBPα-deletion model (Figure 4A). To validate these RNA array results, the *O*-glycosylation level as well as the pertinent enzymes, OGT and OGA, were analyzed using an HCC panel in vivo. The abundance of *O*-GlcNAc was elevated in HCC compared with the corresponding non-tumor sample; meanwhile, a significant reduction in OGA and unchanged OGT were detected in HCC. Interestingly, decreased CEBPα expression was confirmed in HCC (Figure 4B,C). It was thus tempting to speculate that the deregulation of the special glycosidase OGA might be the decisive factor in the OGT/OGA imbalance triggering hyper-*O*-glycosylation. We then attempted to investigate how OGA could be transcriptionally regulated. The Jasper database showed that the OGA gene promoter contains two binding sites for the transcriptional factor CEBPα (Figure 4D). ChIP results showed that CEBPα could pull down fragments of DNA encoding OGA, and CEBPα depletion resulted in a significantly decreased OGA signal (Figure 4E). The results of a luciferase reporter assay in an HEK293T cell model confirmed the binding of CEBPα in the OGA promoter and its positive transcriptional pattern (Figure 4F). Increased OGA protein expression and a reduced *O*-GlcNAc level upon CEBPα overexpression were detected, while the silencing of CEBPα had the reverse effects. The OGT level remained unchanged upon altering CEBPα (Figure 4G), as did the OGT luciferase intensity (Supplementary Figure S4). These findings provide substantial evidence that CEBPα has a significant impact on OGA activation, inhibiting *O*-glycosylation.

### 2.5. RANBP2-Associated OGA Modulation Is Transcriptionally Regulated and Independent of SUMOylation

CEBPα is indicated as a potential SUMO target of RANBP2, and its pertinent role in OGA regulation is proven by the above results. As RANBP2 is a known SUMO E3 ligase, the modulation of the related *O*-GlcNAc enzymes, OGT and OGA, by RANBP2 were then investigated. Western blotting showed a remarkable *O*-GlcNAc decrease and OGA upregulation upon RANBP2 knockdown, while OGT protein expression was unchanged (Supplementary Figure S5A). Interestingly, we found three times as much OGA mRNA in RANBP2-depleted HCC cells, and OGT mRNA remained unaltered by RANBP2 (Supplementary Figure S5B). The subtle OGA mRNA regulation was additionally confirmed by an RNA stability assay, suggesting that RANBP2 did not affect the half-life of OGA mRNA (Supplementary Figure S5C). Bioinformatics analyses showed relatively low scores for predicted SUMO binding sites in both OGT and OGA. Compared with the basal SUMO levels for these two proteins, the SUMO1 bands of OGT and OGA in RANBP2-knockdown HCC cells were not changed. Additionally, no protein of RANBP2 was co-immunoprecipitated by either OGT or OGA (Supplementary Figure S6). These observations indicate that RANBP2 is unlikely to modulate the kinetics of *O*-GlcNAc enzymes by either direct interaction or SUMOylation approaches. The changes in the levels of OGA caused by RANBP2 are due to its transcriptional regulation rather than mRNA decay.

### 2.6. RANBP2 Promotes CEBPα-Dependent O-glycosylation Imbalance in HCC

**Figure 5:**
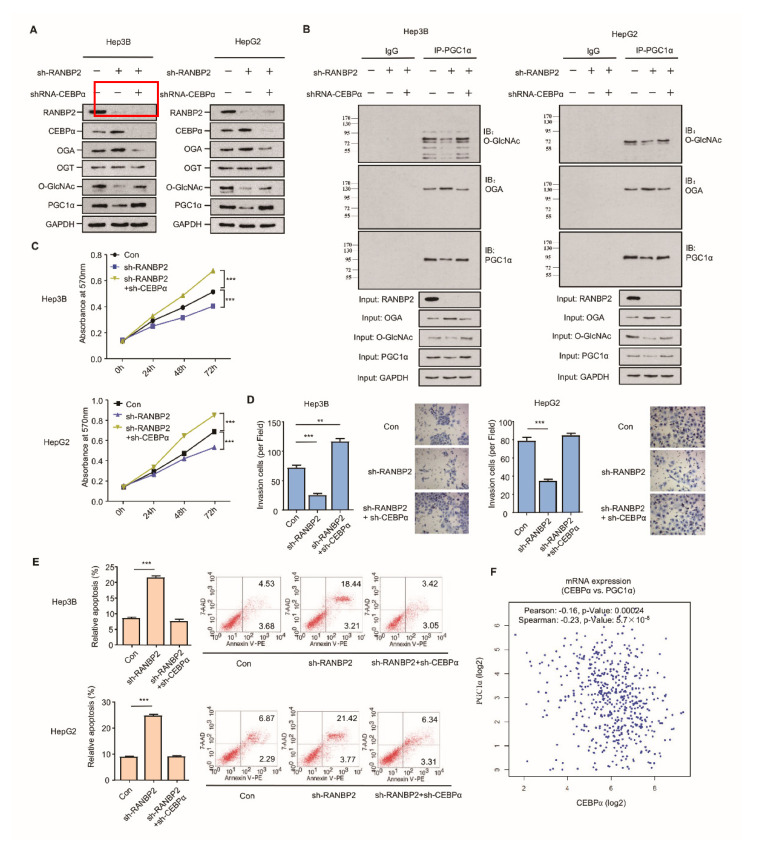
CEBPα is indispensable for RANBP2-associated O-glycosylation imbalance and HCC malignant phenotype in vitro. (**A**,**B**) Western blotting (**A**) and co-immunoprecipitation (**B**) results for the O-glycosylation-related factors and HCC oncogenic PGC1α in response to alterations of RANBP2 and CEBPα levels. Silencing of RANBP2 significantly reduced the total levels of *O*-GlcNAc, accompanied by augmented OGA. Specifically, the acknowledged oncogene PGC1α was O-glycosylation-modified, and it was reduced at the protein level under RANBP2 depletion. The above tendencies were largely reversed by CEBPα knockdown. Notably, a protein interaction between OGA and PGC1α was confirmed. (**C**–**E**) Proliferation (**C**), apoptosis (**D**), and invasion ability (**E**) of HCC cells in response to alterations in RANBP2 and CEBPα expression. Data are presented as the mean ± SD values from three biological replicates. ** *p* < 0.01 and *** *p* < 0.001, *t*-test. (**F**) CEBPα mRNA expression positively correlated with PGC1α expression in HCC (linear regression). Data were obtained from the GEPIA database.

RANBP2 may mainly promote HCC malignancy by regulating the *O*-GlcNAc-associated enzyme OGA; however, whether CEBPα is a crucial intermediate in RANBP2-associated aberrant *O*-glycosylation needs to be further investigated. The silencing of RANBP2 resulted in elevated CEBPα and OGA but less *O*-GlcNAc, results that are in line with our previous findings. Loss-of-function experiments revealed that CEBPα depletion contributed to the low level of the OGA protein and the recovery of *O*-GlcNAc, even in the absence of RANBP2. PGC1α, recognized as a crucial downstream effector in HCC malignancy in terms of mitochondrial biogenesis, tumor transformation, and metabolism, was introduced as an indicator of HCC malignancy [25,26]. PGC1α remained abundant under the double knockdown of RANBP2 and CEBPα (Figure 5A). In particular, a decrease in the *O*-GlcNAc of PGC1α was evident under RANBP2 depletion, which was reversed by further CEBPα knockdown. This phenomenon was also consistent with a previous report showing that *O*-GlcNAcylation enhanced PGC1α stability [9]. It was beyond our expectation that co-immunoprecipitation confirmed the endogenous interaction between OGA and PGC1α. RANBP2 knockdown might facilitate the recruitment of OGA to the dynamically *O*-GlcNAcylated PGC1α, thereby destabilizing PGC1α. CEBPα silencing largely abolished the effect of RANBP2 knockdown, indicating its indispensable role in this hypothetical process (Figure 5B). In addition, both Hep3B and HepG2 cells treated with sh-RANBP2 displayed significantly lower rates of proliferation, reduced invasive ability, and incremental apoptosis. The above changes could also be reversed by sh-CEBPα to a great extent (Figure 5C,D). It is noteworthy that sh-RANBP2 induced apoptosis, which was rescued by sh-CEBPα (Figure 5E). In agreement with these data showing that CEBPα acted as a tumor suppressor in HCC carcinogenesis, its mRNA expression positively correlated with PGC1α expression in patients with HCC (Figure 5F).

Taken together, these observations suggest that the downregulation of CEBPα is essential for RANBP2-associated *O*-glycosylation imbalance and the HCC malignant phenotype in vitro with respect to higher proliferation and invasion as well as less apoptosis.

### 2.7. Silencing of CEBPα Is Essential for RANBP2-Mediated Aberrant O-GlcNAcylation and HCC Tumor Growth In Vivo

**Figure 6:**
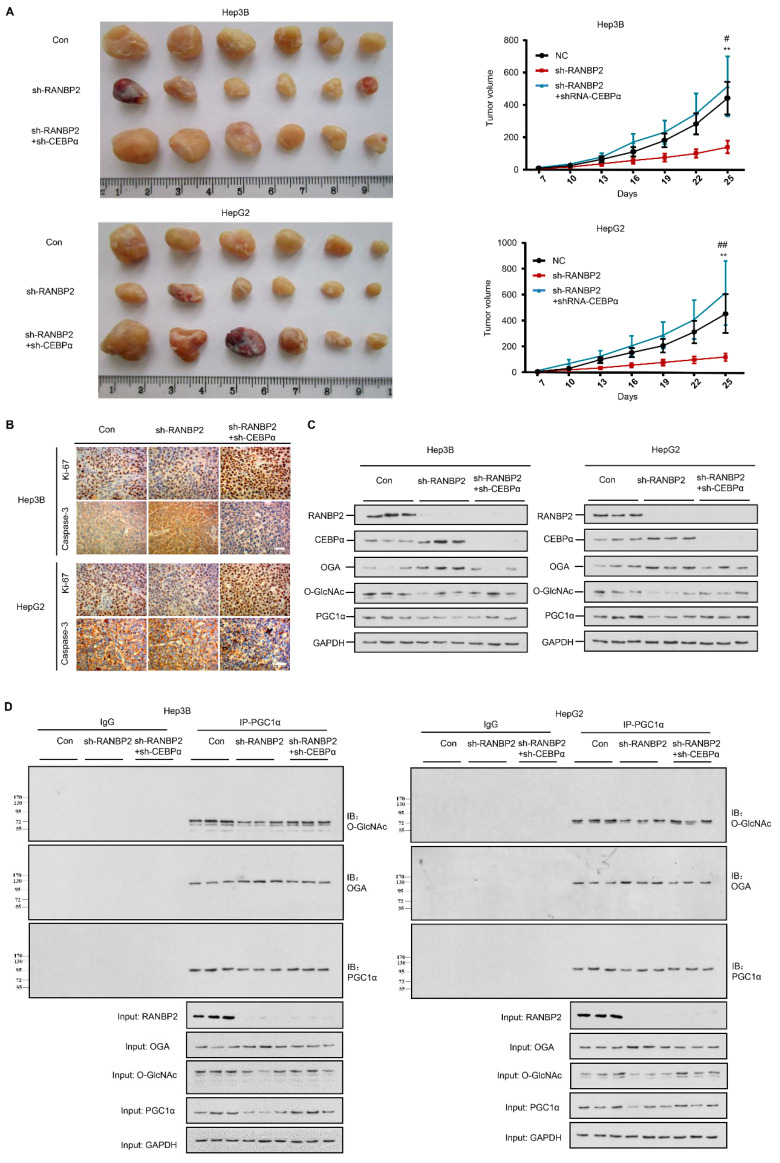
RANBP2 triggers HCC tumorigenicity via the CEBPα-associated imbalance of O-glycosylation homeostasis in vivo. (**A**) Silencing of RANBP2 retarded HCC tumor growth, which was effectively counteracted by CEBPα depletion. The sizes of Hep3B and HepG2 tumors formed in the mice with subcutaneous implantation were monitored every three days. Data are presented as the mean ± SD values (n = 6). sh-RANBP2+sh-CEBPα group: * vs sh-RANBP2, ** *p* < 0.01; *#* vs NC, *#* *p* < 0.05, *##* *p* < 0.01 (*t*-test). (**B**) HCC tumorigenicity was confirmed by the immunohistochemical staining of the isolated subcutaneous tumor tissue. sh-RANBP2 significantly decreased the proliferating marker Ki-67, while it increased the apoptosis marker caspase-3. sh-CEBPα was demonstrated to have opposite effects. Scale bars, 20 mm. (**C**,**D**) Downstream effectors of RANBP2 and CEBPα related to O-GlcNAc modification and tumor promoter PGC1α were tested by immunoblotting (**C**) and co-immunoprecipitation (**D**) in xenograft models.

A RANBP2-induced OGT/OGA imbalance is critical for HCC malignant transformation based on our above findings. As the downregulation of CEBPα is prominent in HCC tumors, we next evaluated the in vivo behavior of CEBPα and the proposed molecular basis in response to RANBP2 using xenograft models. As anticipated, the depletion of CEBPα compensated for the loss of tumorigenic ability in the absence of RANBP2 (Figure 6A). In line with tumorigenic capacity, we found a marked increase in the proliferating marker Ki-67 but attenuation of the apoptosis marker caspase-3 under CEBPα silencing (Figure 6B). The expression tendencies for OGA and PGC1α along with the subsequent O-glycosylation events were in line with the previous in vitro results (Figure 6C,D). Altogether, the above results consolidate the molecular mechanism of RANBP2-associated HCC malignancy, whereby its downstream SUMO target CEBPα is fundamental for OGA inactivation and subsequent hyper-*O*-GlcNAcylation.

## 3. Discussion

Abnormalities in energy metabolism are considered key factors in the natural history of glycosylation in HCC. Notably, hyper-*O*-GlcNAcylation is identified as a “nutrient sensor” that is associated with different metabolic disorders [27]. Accordingly, several solid and non-solid cancers exhibit increased *O*-GlcNAcylation and elevated levels of OGT, which are positively related to higher tumor aggressiveness and poor prognosis [28]. In this study, we found that a rapid increase in *O*-GlcNAcylation occurs in HCC. There are specific mechanisms regulating the levels of *O*-GlcNAcylation. Previous reports and our own consistent clinical data led us to assess OGT/OGA imbalance with a detailed screening, and a decrease in OGA expression at the stage of RANBP2 expression appears to be one of the mechanisms. Gain-of-function studies firstly proved that RANBP2 elevates *O*-GlcNAc via the downregulation of OGA instead of OGT activation. Despite the multiple biological functions of RANBP2 as an element of the nuclear pore complex (NPC), we precluded the redistribution of OGT and OGA based on nuclear–cytoplasmic shuttling by RANBP2. In addition, the direct SUMO-tags were not found in either OGT or OGA. When performing the PCR tests using the above in vitro models, it was, however, intriguing that the OGA mRNA level was affected by RANBP2. These results collectively hint at possible transcriptional regulation by RANBP2, identifying CEBPα as the prominent intermediate.

CEBPα was the first transcription factor identified in the CEBP transcription factor family. Previous studies have suggested that CEBPα is a master transcription factor that reverses liver dysfunction across various liver disease models, including fibrosis, cirrhosis, and HCC [29,30]. HCC appears to develop more rapidly in transgenic CEBPα-knockout models [31]. Increasing the transcriptional activity of CEBPα using short-activating RNA (saRNA) promoted the reversal of HCC development in a rat model, and it is currently being tested in a phase I study as a novel therapeutic oligonucleotide [32]. Recent studies have shown that HCC is caused by the de-differentiation of hepatocytes into cancer stem cells; the dephosphorylation of the tumor suppressor CEBPα at Ser193 or the mutation of Ser193 to Ala results in this process [21]. Our new findings show the fine tuning of OGA in HCC development by RANBP2 through the inactivation of CEBPα, and its function as a tumor suppressor is accordant with clinically relevant reports [33]. However, the opposite observations were made amongst HCC patients, where higher CEBPα mRNA was observed in tumor versus adjacent normal tissue sections [30]. These discrepancies are partly due to the detection of two main isoforms (the p42 active form and p30 inactive form) that have opposite functions upon post-transcriptional regulation. Notably, the homologous CEBPβ is significantly downregulated by RANBP2, but it is not a SUMO target of RANBP2. Although the pertinence of the role of CEBPβ in HCC tumorigenesis is controversial, the mechanism by which RANBP2 modulates CEBPβ in HCC tumorigenesis will be intriguing to decipher.

SUMO is crucial for various types of cancer due to its reversible nature in cell cycle progression and genome integrity [34]. Apart from RANBP2, multiple SUMO enzymes are responsible for cancer progression and represent targetable cancer metabolic biomarkers [35,36]. In the current study, the immunofluorescence study indicated that CEBPα mostly resides in nuclei as a typical transcriptional factor. According to our observations, endogenous RANBP2 reduces the nuclear intensity of CEBPα, and at the same time, the SUMOylation of CEBPα promotes its instability and degradation in the proteasomal pathway that generally occurs in the cytosol. It is intriguing that RANBP2 might inhibit CEBPα’s transcriptional activity through both NPC and SUMOylation properties. In addition to the known cascade of SUMO signaling, which comprises SUMO1 (activation) → SUMO2 (conjugation) → SUMO3 (ligation), the finding more intriguing for us is that the SUMO E3 ligase RANBP2 could also trigger the upstream SUMO1 process, possibly because RANBP2 mainly locates at the nuclear pore and helps to form the nuclear pore complex encompassing multiple SUMO1 enzymes. Therefore, we speculate that RANBP2 may facilitate the nuclear export or impede the import of CEBPα under the control of SUMO tagging. The potential effects of RANBP2 on the CEBPα nucleocytoplasmic transport machinery greatly attract our interest. Furthermore, based on the mutual interaction of the two molecules, whether CEBPα forms a complex with RANBP2 at the nuclear pore will be intriguing to elucidate.

The critical effect of OGA on the blockade of *O*-GlcNAcylation is mediated by CEBPα activation. As a glycosidase, OGA participates in the transient *O*-GlcNAc modification of substrates, including PGC1α. The results of the current study surprisingly unravel the interaction of OGA and PGC1α. These two proteins are reported to be distributed in both the nucleus and cytoplasm, and it is conceivable that their structural enzyme–substrate effect may provide more insights into the molecular mechanism by which *O*-GlcNAc improves the outcomes of HCC therapy.

## 4. Materials and Methods

### 4.1. Cell Lines and Cell Culture

The human HCC cell lines Hep3B and HepG2 were purchased from the American Type Culture Collection (ATCC, Manassas, VA, USA). All of these cell lines were continuously cultured in Dulbecco’s Modified Eagle Medium (Sigma-Aldrich, St. Louis, MO, USA) containing 10% fetal bovine serum (FBS) (Gibco, New York, NY, USA), 100 IU/mL penicillin G, and 100 μg/mL streptomycin (Sigma-Aldrich, St. Louis, MO, USA). The cell lines were incubated at 37 °C under a humidified atmosphere with 5% CO_2_.

### 4.2. Plasmid Construction, RNA Interference, and Transfection

The full-length pEF-HA-RanBP2 plasmid was gifted by Dr. Melchior (University of Heidelberg) and Dr. Kehlenbach (University of Göttingen). A CEBPα plasmid tagged with and without GFP as well as a SUMO1 plasmid tagged with HIS was purchased from Genechem Biotechnology Company (Shanghai, China). The primer information is listed in Supplementary Table S2.

The short hairpin RNA (shRNA) for RANBP2, CEBPα, and their corresponding negative controls was synthesized by Genechem (Shanghai, China). The target sequences for RANBP2 and CEBPα are shown in Supplementary Table S2. The transfections were conducted using Lipofectamine 2000 (Invitrogen) according to a previously described protocol [37]. The knockdown efficiencies were assessed by immunofluorescence and Western blotting, and an RNAi sequence with relatively high results was selected for further experimentation.

### 4.3. Reagents

The chemical reagents 2-D08 (2′,3′,4′-trihydroxy flavone, an inhibitor of protein SUMOylation; cat. #s869601) and MG132 (a proteasome inhibitor; cat. #s2619) were obtained from Selleckchem (Houston, TX, USA). CHX (cycloheximide, a protein synthesis inhibitor; cat. #2112s) was obtained from Cell Signaling Technology (Beverly, MA, USA). Streptonigrin (a SUMO agonist, known to be an inhibitor of the SUMO-specific protease SENP1; CAS no. 3930-19-6) was obtained from Santa Cruz (Dallas, TX, USA).

### 4.4. Mutagenesis

The mutation and truncation of CEBPα were performed using PCR-based methods. The CEBPα CDS (NM_006706.4) was synthesized and subcloned into the vector pEGFP-C1 (Clontech, 5′ BglII-3′ BamHI). A point mutation in Lys196 was introduced to histidine (H); then, WT CEBPα and mutants were validated by DNA sequencing and Western blotting with help from Kangchen Biotech (200233; Shanghai, China).

### 4.5. Cell Viability, Apoptosis, and Invasion Assay

Cell proliferation was analyzed using a commercial CCK-8 assay kit (#C0038, Beyotime). Fluorescence-activated cell sorting (FACS) was used to assess apoptosis with the Annexin V-FITC/PI staining kit (Mbchem). Cell invasion was assessed by the Transwell assay with a 6-well insert device (8 μm pore size; Corning Life Sciences, Bedford, MA, USA) and Biocoat Matrigel (BD Biosciences) according to the manufacturer’s instructions.

### 4.6. Luciferase Assay

Cells were seeded into 96-well plates and grown to approximately 80% confluency on the second day. Next, the relevant reporter plasmids and the PRL-TK reporter were transiently co-transfected into the cells. After 48 h, the firefly luciferase activity and Renilla activity were determined. The ratio of firefly luciferase activity to Renilla activity was regarded as indicating the activity of the gene promoters. Each experiment was performed in triplicate.

### 4.7. Cycloheximide (CHX)-Based Protein Stability Assay

Cells were treated with 10 μM cycloheximide (CHX) for the indicated periods (0, 1, 2, 4, and 8 h) to block protein synthesis. A concentration of 20 μM MG132 was also administered to inhibit the proteasome before harvest. Crude extracts were prepared, and CEBPα protein expression was then assayed as described previously [38].

### 4.8. mRNA Decay Assay

To measure mRNA stability, 5 g/mL actinomycin D (Sigma Aldrich, St. Louis, MO, USA) was added to cells to inhibit transcription, followed by incubation for the different times indicated. Total RNA was extracted at each time point and quantitated by RT-PCR. The transcript levels were plotted to create the appropriate nonlinear regression curves using a one-phase decay equation. Exponential fitting curves were determined to quantify the RNA decay rate constant (y = a*e-kt; where k is the decay rate constant, y is the relative amount of RNA, and t is the time). The rate of mRNA turnover was estimated according to a previously published paper and half-life t1/2 = ln(2)/k [39]. The transcript 18 s rRNA, which does not decay over time, was detected as a control.

### 4.9. Immunohistochemistry

Tissue samples were obtained from the specimens of patients who met the following criteria: (1) informed consent was provided; (2) the patients were clinically and pathologically diagnosed with HCC; (3) the patients did not undergo any neoadjuvant therapy before surgery; (4) adjacent non-neoplastic liver tissues were available for comparison. A total of 54 HCC specimens embedded in paraffin were cut into 5-micrometer sections. The sections were dewaxed in xylene, rehydrated with a gradient ethanol series, treated with 0.3% H_2_O_2_ in methanol, blocked with 1% protein blocking agent (PBA), and incubated with primary antibody at 4 °C overnight. After the addition of a polymer enhancer, the sections were treated with peroxidase-labeled streptavidin for 30 min at room temperature. The antibody reaction was visualized using a fresh substrate solution containing diaminobenzidine. The sections were counterstained with hematoxylin, dehydrated, and mounted in glycerol–vinyl alcohol.

The degree of immunostaining of the formalin-fixed, paraffin-embedded sections was reviewed and scored separately by two independent pathologists who were blinded to the histopathological features of the samples. Based on the proportions of tumor cells, the following scores were assigned: 0, no tumor cells; 1, <10% tumor cells; 2, 10–35% tumor cells; 3, 35–75% tumor cells; and 4, >75% tumor cells. The staining intensity was scored as follows: 1, no staining; 2, weakly stained (light yellow); 3, moderately stained (yellow brown); and 4, strongly stained (brown). The staining index was determined by multiplying the staining intensity score by the tumor cell proportion score (high expression, ≥6; low expression, <6).

### 4.10. Immunofluorescence Staining

Cells were grown on polyethyleneimine-coated coverslips, washed with pre-warmed phosphate-buffered saline (PBS), fixed in 4% paraformaldehyde for 15 min, permeabilized with 0.5% TritonX-100 in PBS for 10 min, blocked with 3% bovine serum albumin (BSA) solution for 1 h, and incubated with anti-primary antibodies in 3% BSA at 4 °C overnight (the antibody information is listed in Supplementary Table S3). The cells were then rinsed three times for 5 min with PBS and then incubated individually with primary antibodies in 3% BSA at 37 °C for 90 min. Alexa488 or Alexa555 anti-rabbit secondary antibodies (1:100, Invitrogen) were added for 1 h in the dark. Nuclei were counterstained with DAPI (1:1000, sc-3598; Santa Cruz) in PBS at room temperature for 2 min, rinsed with PBS three times for 3 min, and mounted with SlowFade Gold Antifade Reagent (S36942, Life Technologies). Images were obtained from a confocal microscope (TCSSP8, Leica Microsystems) equipped with an acousto-optic beam splitter, a 405-nanometer laser (for DAPI), an argon laser (488 nm for Alexa 488), and a diode-pumped solid-state (DPSS) laser (561 nm).

### 4.11. Transcriptional Factor/DNA Array

Nuclear extracts of Hep3B cells treated with RANBP2 overexpression and empty plasmids were subjected to the TranSignalTM Protein/DNA Array (Panomics, Redwood City, CA, USA) according to the manufacturer’s instructions.

### 4.12. Quantitative Real-Time RT-PCR and RNA Array

Quantitative real-time RT-PCR (qRT-PCR) analyses were carried out with the conventional method. The primers used for the qPCR of the referenced genes are shown in Supplementary Table S2. A high-throughput RNA array using sh-CEBPα transfection in Hep3B cells was performed with the support of Kangchen Biotech.

### 4.13. Chromatin Immunoprecipitation (ChIP) Assay

For the ChIP assay, 1.5 × 10^6^ cells were subjected to a two-step dual cross-linking procedure based on previously described methods [40]. In brief, protein–DNA complexes were immunoprecipitated with anti-CEBPα antibodies. Purified DNA was added to agarose gel and analyzed by qPCR using a BioRad CFX-96 quantitative thermocycler and a SsoFast EvaGreen Low-ROX qPCR SuperMix (BioRad). The primer pairs used for qPCR quantification are listed in Supplementary Table S2. The data were analyzed according to the ΔΔCT method (Applied Biosystems).

### 4.14. Protein Extraction, Immunoprecipitation, and Western Blot Analysis

Protein extracts were obtained from fresh cells once with PBS and in RIPA buffer (Thermo Scientific, Waltham, MA, USA) supplemented with protease inhibitor and phosphatase inhibitor (Roche, Welwyn Garden, Switzerland).

Immunoprecipitation was performed using protein G-agarose (Millipore, Temecula, CA, USA). Supernatants (1–2 mg of total protein in 1 mL) were incubated with 2 μg of primary antibody, and then, the bound proteins were eluted in SDS sample buffer and analyzed by SDS–PAGE and immunoblotting.

For Western blot analysis, primary antibodies were used, as indicated in Supplementary Table S3. The blots were then developed with ECL Western blotting reagents (Pierce Biotechnology, Rockford, IL, USA). The signal intensity was quantified with ImageJ (National Institutes of Health, Bethesda, MD, USA).

### 4.15. Tumor Xenograft Models

Five-week-old male BALB/c nude mice were randomly divided into six groups. Specifically, xenograft tumors were made using subcutaneous inoculation in the right axillary fossa with 200 μL (1 × 10^6^ cells) of sh-RANBP2 and sh-CEBPα in Hep3B/HepG2 cells and control cells. The sizes of the palpable tumors were recorded every 3 days by measuring the tumor length (L, the longest diameter) and width (W, the shortest diameter), which were recorded six consecutive times. All the mice were sacrificed after 35 days. The tumor volume (V) was calculated according to the formula V = 1/2 (L × W²).

### 4.16. Bioinformatic Analyses

Protein–protein structural interactions were predicted using the PHYRE2 (http://www.sbg.bio.ic.ac.uk/phyre2/, accessed on 1 June 2019) website and Discovery Studio software. The survival, differential expression, and correlation of the candidate genes were assessed using the Gene Expression Profiling Interactive Analysis (GEPIA) database (http://gepia.cancer-pku.cn, accessed on 1 February 2020), the starBase Pan-Cancer Analysis Platform (http://starbase.sysu.edu.cn/panCancer.php, accessed on 1 February 2020), the Cancer Genome Atlas (TCGA) database (https://cancergenome.nih.gov/, accessed on 1 February 2020), the Cancer RNA-Seq Nexus (CRN) database (http://syslab4.nchu.edu.tw/, accessed on 1 February 2020), and NCBI’s Gene Expression Omnibus database (https://www.ncbi.nlm.nih.gov/geo/, accessed on 1 February 2020). GPS-SUMO (http://sumosp.biocuckoo.org/, accessed on 1 June 2019) was applied to predict the SUMO sites. The specific binding of transcriptional factors in gene promoters was predicted using the Jasper database (http://jaspar2016.genereg.net/, accessed on 1 June 2019). Gene set enrichment analysis (GSEA) for RANBP2-relevant gene signatures was performed with the GSEA software v.2.0 according to a reported protocol [41].

### 4.17. Statistical Analysis

Statistical analyses were performed using the SPSS 19.0 software (version 19.0; SPSS Inc., Chicago, IL, USA). The results are presented as the mean ± SD, and two-group comparisons were evaluated using Student’s *t*-test.

The relationships between RANBP2 expression and the clinicopathological characteristics were analyzed using the χ^2^ test. Based on the correlation between the IHC score and patients’ survival, the cut-off point for each dataset subgroup was determined using the survminer R package. The “surv-cut point” function, which repeatedly tested all the potential cut points to find the maximum rank statistic, was applied to the IHC score. The patients were then divided into high- and low-score groups based on maximally selected log-rank statistics to reduce the batch effect of calculation. In the univariate survival analysis, cumulative survival curves were calculated according to the Kaplan–Meier method, and the survival curves were analyzed using the log-rank test.

## 5. Conclusions

In summary, our data not only reveal the key role of RANBP2 in *O*-GlcNAc modification and HCC development, but also identify CEBPα as its interacting transcriptional factor and SUMOylation target. The inactivation of CEBPα by RANBP2 is required for OGA downregulation and subsequent hyper-*O*-GlcNAcylation. These findings suggest that the pharmacological manipulation of the RANBP2–CEBPα–OGA pathway may represent a potential strategy for HCC treatment.

## Data Availability

The data presented in this study are available in Supplementary Materilas.

## Supplementary Materials

The following are available online at https://www.mdpi.com/2072-6694/13/14/3475/s1, Figure S1: RANBP2 is enriched in HCC and indicates poor prognosis. (A) RANBP2 mRNA level comparison between HCC and adjacent non-tumor tissue from the GEO databases (GSE33006, GSE46408). * *p* < 0.05 and *** *p* < 0.001, *t*-test. (B) Survival curve from clinical samples showed that RANBP2 was significantly associated with shorter survival in our HCC patients (*n* = 50). Among total 54 patients, 50 cases having follow-up data were selected, Figure S2: Western blotting results of RANBP2 (A) and CEBPα (B) in response to their different shRNA. The sequence of sh3-RANBP2 and sh1-CEBPα obtained the relatively high knockdown efficiency (RT-qPCR not shown), which were selected for further experiment, Figure S3: The expressional tendencies of endogenous CEBPα and CEBPβ by immunofluorescence staining. Merge, RANBP2+ CEBPα+DAPI; Scale bars, 20 µm, Figure S4: Luciferase reporter assay reveals no transcriptional regulation of OGT by CEBPα. The ratio of firefly luciferase activity to Renilla activity was indicated as gene promoter activities. Each experiment was performed in triplicate, Figure S5: RANBP2 promotes *O*-glycosylation via downregulating OGA transcription while not affecting OGT expression. (A) The remarkable *O*-GlcNAc decrease and OGA upregulation were confirmed in the presence of RANBP2 knockdown, while OGT protein expression was unchanged. (B) RANBP2 negatively regulated transcriptional activity of OGA other than OGT. Profiles of OGT and OGA mRNA levels were implicated in sh-RANBP2 Hep3B and HepG2 cells compared to the controls. (C) Measurement of the RNA stability of OGA by RT-qPCR in presence of the transcriptional inhibitor Actinomycin D (ActD) at indicated time points. Half-life of OGA mRNA (t_1/2_) in both cells was calculated from each experiment shown in the graph. 18S rRNA was conducted as an internal control, Figure S6: RANBP2 associated OGA modulation is independent of SUMOylation. Co-immunoprecipitation showed no protein interactions of OGT-RANBP2 or OGA-RANBP2. The non-changed global SUMO levels of OGT and OGA upon RANBP2 knockdown were also detected using anti-SUMO1 antibody, Table S1. Correlation between RANBP2 expression and clinicopathologic characteristics of the hepatocellular carcinoma patients. Percentage values are shown in parentheses, Table S2. Primers and shRNA used in this study, Table S3: Antibodies for western blot, immunoprecipitation, immunofluorescence, and immunohistochemistry in this study.

## Abbreviations

- **HCC:** Hepatocellular carcinoma
- **RANBP2:** RAN-binding protein 2
- **SUMO:** Small ubiquitin-like modifier
- **NPC****:** Nuclear pore complex
- **CEBPα:** CCAAT/enhancer-binding protein alpha
- **CEBPβ:** CCAAT/enhancer-binding protein beta
- ***O-*GlcNAc:** *O-*linked N-acetylglucosamine
- **OGT:** *O-*GlcNAc transferase
- **OGA:** *O-*GlcNAcase
- **PGC1α:** Peroxisome proliferative-activated receptor gamma coactivator 1 alpha
- **PTM:** Post-translational modification
- **ChIP:** Chromatin immunoprecipitation
- **IHC:** Immunohistochemistry
- **CHX:** Cycloheximide
- **GSEA:** Gene set enrichment analysis
- **2-D08:** 2′,3′,4′-trihydroxyflavone
- **SN:** Streptonigrin
