# Correction: Liu et al. RANBP2 Activates *O*-GlcNAcylation through Inducing CEBPα-Dependent OGA Downregulation to Promote Hepatocellular Carcinoma Malignant Phenotypes. *Cancers* 2021, *13*, 3475

**DOI:** 10.3390/cancers16051018

**Published:** 2024-02-29

**Authors:** Xiaoming Liu, Xingyu Chen, Mengqing Xiao, Yuxing Zhu, Renjie Gong, Jianye Liu, Qinghai Zeng, Canxia Xu, Xiong Chen, Fen Wang, Ke Cao

**Affiliations:** 1 Department of Oncology, Third Xiangya Hospital of Central South University, Changsha 410013, China; 2 Department of Gastroenterology, Third Xiangya Hospital of Central South University, Changsha 410013, China; 3 Department of Urology, Third Xiangya Hospital of Central South University, Changsha 410013, China; 4 Department of Dermatology, Third Xiangya Hospital of Central South University, Changsha 410013, China

In the original publication [1], there was a mistake in Figure 6 as published. We mistakenly exported the wrong IHC results for the “Caspase-3 in HepG2 cell line” shown in Figure 6B. The corrected Figure 6 appears below.

The authors state that the scientific conclusions are unaffected. This correction was approved by the Academic Editor. The original publication has also been updated.

## Figures and Tables

**Figure 6:**
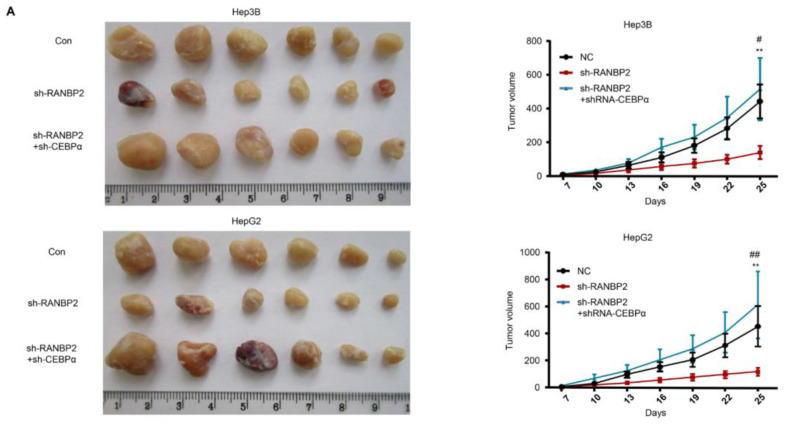

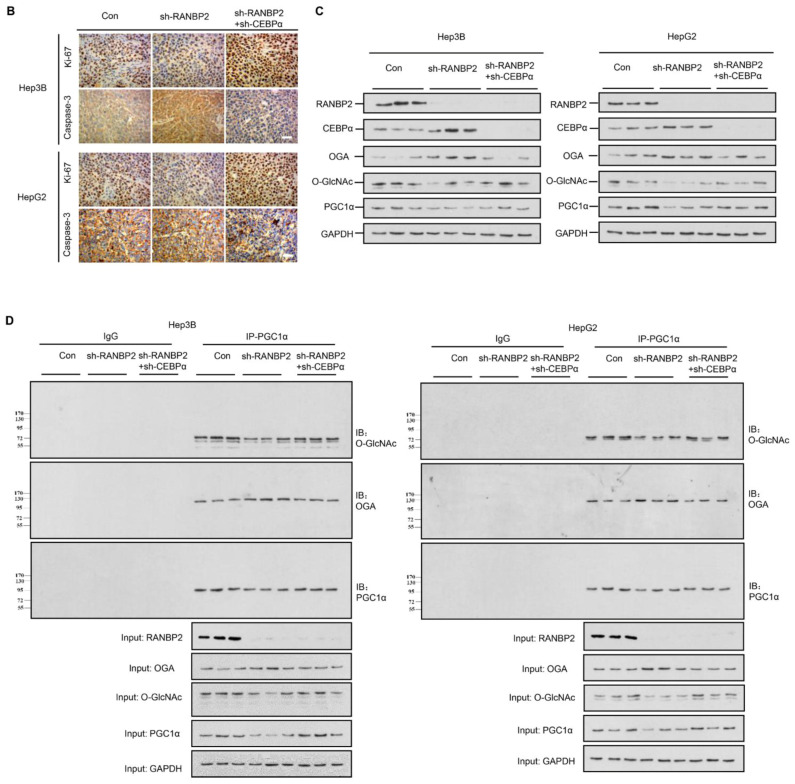
RANBP2 triggers HCC tumorigenicity via the CEBPα-associated imbalance of O-glycosylation homeostasis in vivo. (**A**) Silencing of RANBP2 retarded HCC tumor growth, which was effectively counteracted by CEBPα depletion. The sizes of Hep3B and HepG2 tumors formed in the mice with subcutaneous implantation were monitored every three days. Data are presented as the mean ± SD values (n = 6). sh-RANBP2+sh-CEBPα group: * vs sh-RANBP2, ** *p* < 0.01; *#* vs NC, *#* *p* < 0.05, *##* *p* < 0.01 (*t*-test). (**B**) HCC tumorigenicity was confirmed by the immunohistochemical staining of the isolated subcutaneous tumor tissue. sh-RANBP2 significantly decreased the proliferating marker Ki-67, while it increased the apoptosis marker caspase-3. sh-CEBPα was demonstrated to have opposite effects. Scale bars, 20 mm. (**C**,**D**) Downstream effectors of RANBP2 and CEBPα related to O-GlcNAc modification and tumor promoter PGC1α were tested by immunoblotting (**C**) and co-immunoprecipitation (**D**) in xenograft models.
