# Supplementary material for: RANBP2 Activates O-GlcNAcylation through Inducing CEBPα-Dependent OGA Downregulation to Promote Hepatocellular Carcinoma Malignant Phenotypes
Source: Cancers (Basel). 2021 Jul 12;13(14):3475. doi: 10.3390/cancers13143475 (PMC8304650; doi:10.3390/cancers13143475)
Supplement: Supplementary file 1 [file cancers-13-03475-s001.zip › cancers-1259697-suppl-proof/cancers-1259697-supplementary.pdf]

Supplemental Materials: RANBP2 Activates *O*-GlcNAcylation through Inducing CEBPα-Dependent OGA Downregulation to Promote Hepatocellular Carcinoma Malignant Phenotypes

Xiaoming Liu, Xingyu Chen, Mengqing Xiao, Yuxing Zhu, Renjie Gong, Jianye Liu, Qinghai Zeng, Canxia Xu, Xiong Chen, Fen Wang and Ke Cao

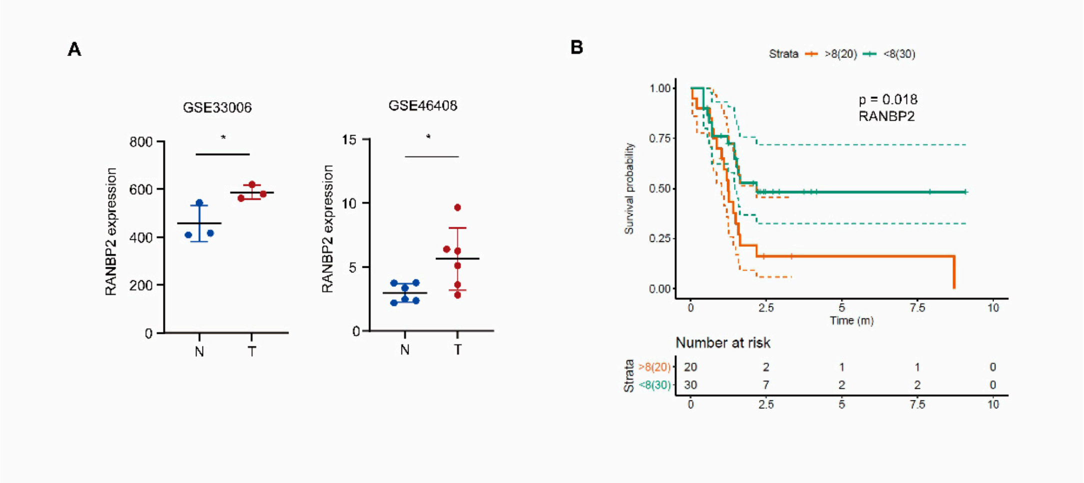

Figure S1. RANBP2 is enriched in HCC and indicates poor prognosis. (**A**) RANBP2 mRNA level comparison between HCC and adjacent non-tumor tissue from the GEO databases (GSE33006, GSE46408). *p < 0.05 and ****p* < 0.001, t-test. (**B**) Survival curve from clinical samples showed that RANBP2 was significantly associated with shorter survival in our HCC patients (*n* = 50). Among total 54 patients, 50 cases having follow-up data were selected.

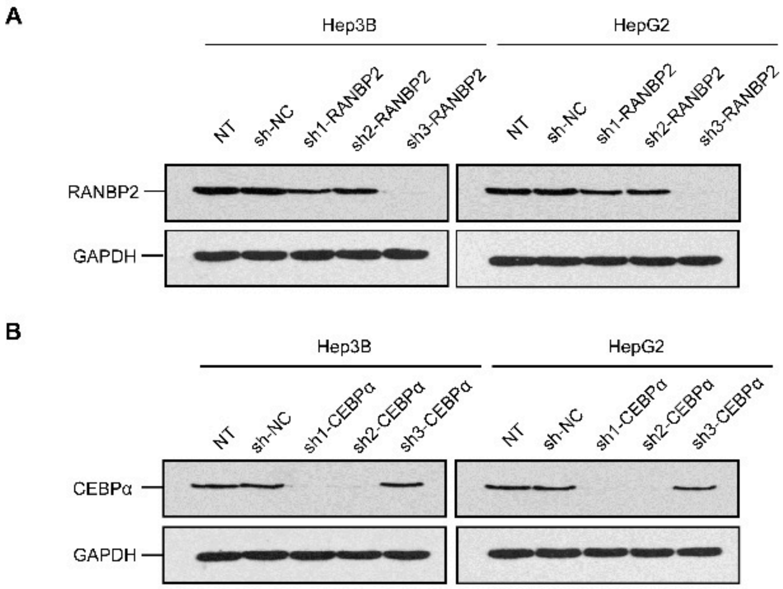

Figure S2. Western blotting results of RANBP2 (**A**) and CEBPα (**B**) in response to their different shRNA. The sequence of sh3-RANBP2 and sh1-CEBPα obtained the relatively high knockdown efficiency (RT-qPCR not shown), which were selected for further experiment.

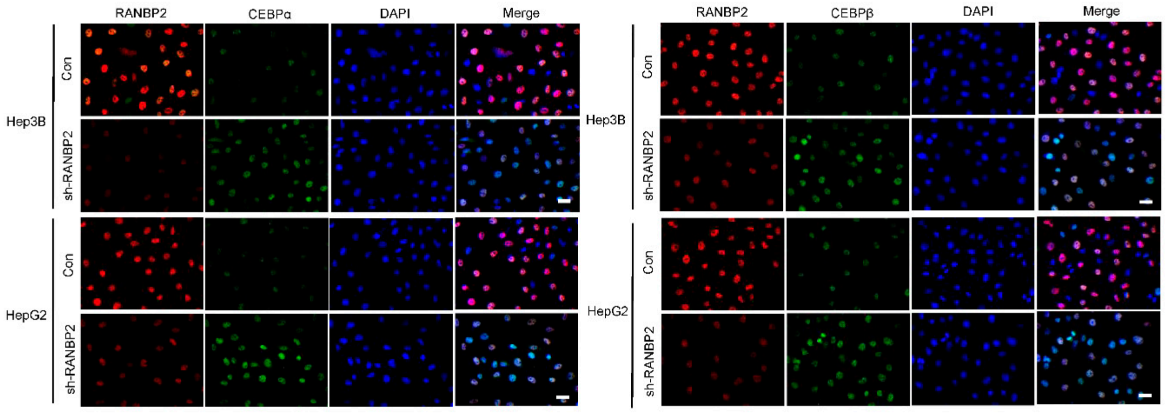

Figure S3. The expressional tendencies of endogenous CEBPα and CEBPβ by immunofluorescence staining. Merge, RANBP2+ CEBPα+DAPI; Scale bars, 20 μm.

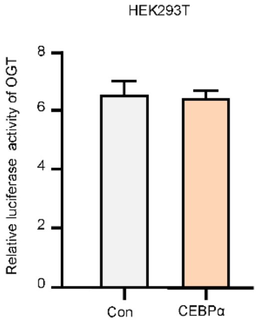

Figure S4. Luciferase reporter assay reveals no transcriptional regulation of OGT by CEBPα. The ratio of firefly luciferase activity to Renilla activity was indicated as gene promoter activities. Each experiment was performed in triplicate.

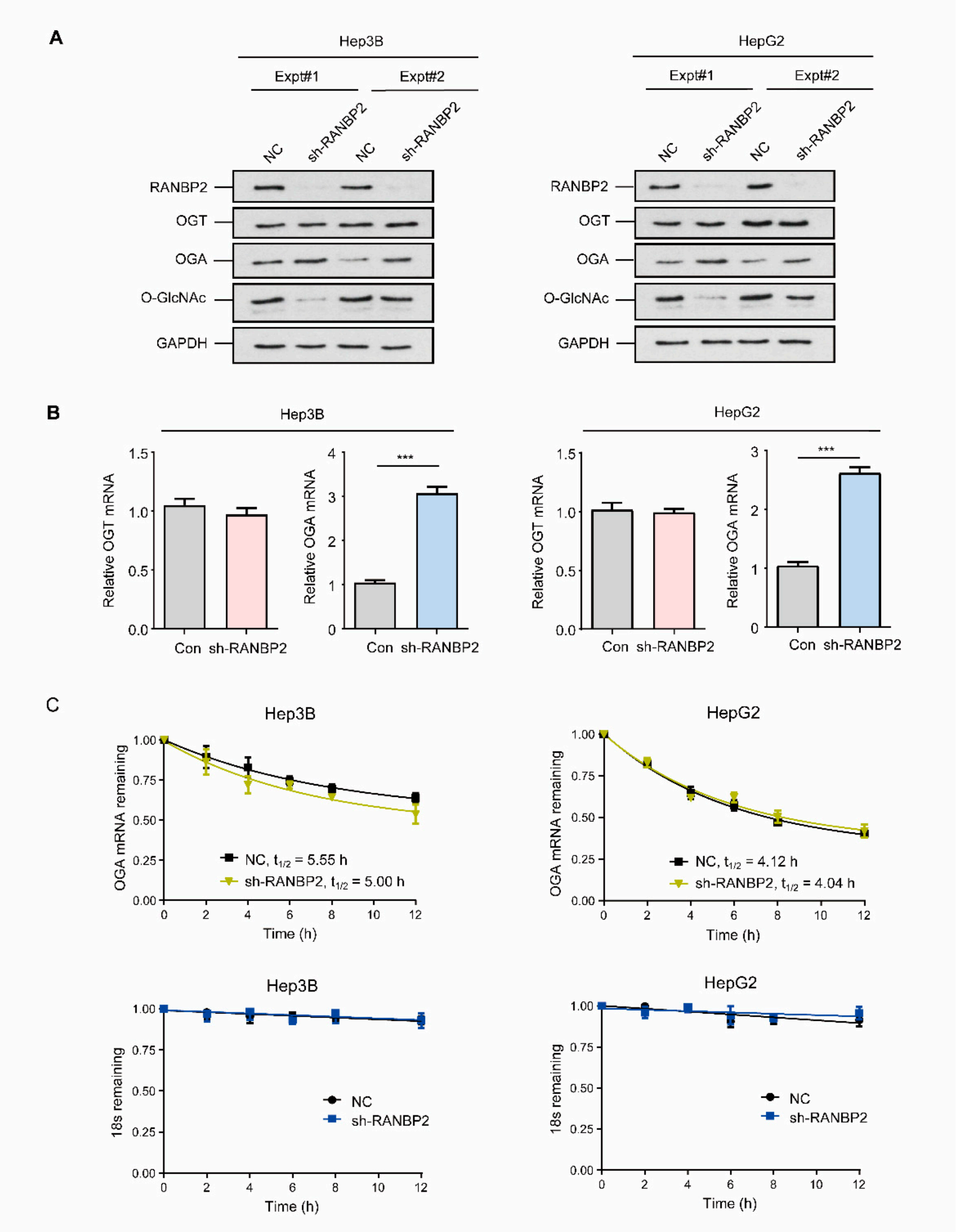

Figure S5. RANBP2 promotes *O*-glycosylation via downregulating OGA transcription while not affecting OGT expression. (**A**) The remarkable *O*-GlcNAc decrease and OGA upregulation were confirmed in the presence of RANBP2 knockdown, while OGT protein expression was unchanged. (**B**) RANBP2 negatively regulated transcriptional activity of OGA other than OGT. Profiles of OGT and OGA mRNA levels were implicated in sh-RANBP2 Hep3B and HepG2 cells compared to the controls. (**C**) Measurement of the RNA stability of OGA by RT-qPCR in presence of the transcriptional inhibitor Actinomycin D (ActD) at indicated time points. Half-life of OGA mRNA ($t_{1/2}$) in both cells was calculated from each experiment shown in the graph. 18S rRNA was conducted as an internal control.

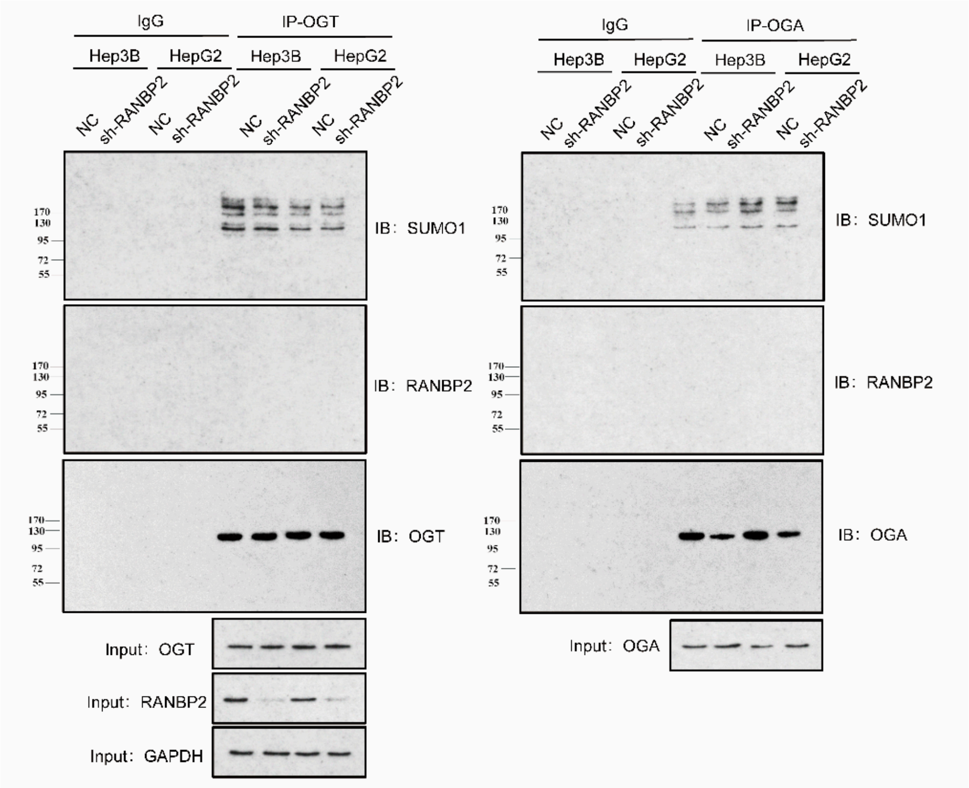

Figure S6. RANBP2 associated OGA modulation is independent of SUMOylation**.** Co-immunoprecipitation showed no protein interactions of OGT-RANBP2 or OGA-RANBP2. The non-changed global SUMO levels of OGT and OGA upon RANBP2 knockdown were also detected using anti-SUMO1 antibody.

Table S1. Correlation between RANBP2 expression and clinicopathologic characteristics of the hepatocellular carcinoma patients. Percentage values are shown in parentheses.

Variables	Total Number	RANBP2 Expression (%)		
		Low expression (n = 21)	High Expression (n = 33)	p-Value
Age (y)				0.147
≤53[a]	25	8 (32.0)	17 (68.0)	
>53	29	13(44.8)	16(55.2)	
Gender				**0.006**
Male	47	15 (31.9)	32 (68.1)	
Female	7	6 (85.7)	1 (14.3)	
Tumor size (cm)				**<0.001**
≤6.7[b]	22	15 (68.2)	7(31.8)	
>6.7	32	6 (18.8)	26 (81.2)	
Tumor multiplicity				0.892
Solitary	38	15 (39.5)	23 (60.5)	
Multiple	16	6(37.5)	10 (62.5)	
Edmondson-Steiner grade				**0.016**
I-II	28	16 (57.1)	12 (42.9)	
III	25	5 (20.0)	20 (80.0)	
IV	1	0 (0)	1(100.0)	
pT classification				**0.042**
PT1/PT2	30	16(53.3)	14(46.7)	
PT3	22	5(22.7)	17 (77.3)	
PT4	2	0 (0.0)	2 (100.0)	
pN classification				0.971
PN0	41	16 (39.0)	25 (61.0)	

PN1	13	5 (38.5)	8 (61.5)

[a] median age. [b] median size. [c] Chi-square test.

Table S2. Primers and shRNA used in this study.

Name	Forward/Sense	Reverse/Anti-Sense
OGT-qPCR	TCCTGATTTGTACTGTGTTCGC	AAGCTACTGCAAAGTTCGGTT
OGA-qPCR	GAAGGAGAGTCAAGCGACGTT	TCCATAACCCAAGGTCTTCCAT
RANBP2-qPCR	AAACCTCCGATTGCAGCTCAT	GGCAAAGATGGCCTTAATCCT
CEBPα-qPCR	CTGCTATAGGCTGGGCTTCC	AGCTGAGGGCAAAGGAGAAC
GAPDH-qPCR	GGAGCGAGATCCCTCCAAAAT	GGCTGTTGTCATACTTCTCATGG
β-actin	CATGTACGTTGCTATCCAGGC	CTCCTTAATGTCACGCACGAT
18S	CAGCCACCCGAGATTGAGCA	TAGTAGCGACGGGCGGTGTG
OGA-qPCR (for CHIP)	GAGGCAGGAGAATAGCCTGA	TTAGCCGTAGCCCAACTTGT
GAPDH-qPCR (for CHIP)	TACTAGCGGTTTTACGGGCG	TCGAACAGGAGGAGCAGAGAGCGA
RANBP2-shRNA-3093	CACCGCTTCAAGATCTGCAGAATCTCT<br>CGAGAGATTCTGCAGATCTTGAAGC	AAAAGCTTCAAGATCTGCAGAATCTCT<br>CGAGAGATTCTGCAGATCTTGAAGC
RANBP2-shRNA-3718	CACCGCGCGAAATTGTTTCGTTTCGCT<br>CGAGCGAAACGAAACAATTTCGCGC	AAAAGCGCGAAATTGTTTCGTTTCGCT<br>CGAGCGAAACGAAACAATTTCGCGC
RANBP2-shRNA-4170	CACCGCAAAGAAAGAAGGGTCTTGGC<br>TCGAGCCAAGACCCTTCTTTCTTTGC	AAAAGCAAAGAAAGAAGGGTCTTGG<br>CTCGAGCCAAGACCCTTCTTTCTTTGC
CEBPα-shRNA-945	CACCGAAGTCGGTGGACAAGAACAGC<br>TCGAGCTGTTCTTGTCCACCGACTTC	AAAAGAAGTCGGTGGACAAGAACAG<br>CTCGAGCTGTTCTTGTCCACCGACTTC
CEBPα-shRNA-951	CACCGGTGGACAAGAACAGCAACGA<br>CTCGAGTCGTTGCTGTTCTTGTCCACC	AAAAGGTGGACAAGAACAGCAACGA<br>CTCGAGTCGTTGCTGTTCTTGTCCACC
CEBPα-shRNA-954	CACCGGACAAGAACAGCAACGAGTA<br>CTCGAGTACTCGTTGCTGTTCTTGTCC	AAAAGGACAAGAACAGCAACGAGTA<br>CTCGAGTACTCGTTGCTGTTCTTGTCC

Table S3. Antibodies for western blot, immunoprecipitation, immunofluorescence, and immunohistochemistry in this study.

Primary Antibodies	Dilution	Company/Catalog
RANBP2	1:1000 (WB); 1:100 (IF); 1:500 (IHC)	Abcam, ab64276
CEBPα	1:500 (WB); 1:100 (IF); 1:50 (IP); 1:200 (IHC)	Abcam, ab15047
CEBPβ	1:1000 (WB); 1:100 (IF); 1:50 (IP); 1:200 (IHC)	Abcam, ab32358
O-GlcNAc	1:1000 (WB); 1:200 (IHC)	Abcam, ab2739
OGT	1:200 (WB); 1:50 (IP); 1:200 (IHC)	Proteintech, 11576-2-AP
OGA	1:200 (WB); 1:50 (IP); 1:200 (IHC)	Proteintech, 14711-1-AP
PGC1α	1:500 (WB); 1:50 (IP)	Novus, NBP1-04676
SUMO1	1:200 (WB)	Proteintech, 10329-1-AP
GFP	1:1000 (WB); 1:100 (IP)	Abcam, ab290
HA	1:2000 (WB)	Abcam, ab9110
His	1:500 (WB)	Abcam, ab18184M
Human IgG	1:150 (IP)	Bioss, #bs-0297P
Caspase-3	1:200 (IHC)	Proteintech, 19677-1-AP
Ki-67	1:100 (IHC)	Genetex, GTX16667
LAMINB1	1:2000 (WB)	Abcam, ab65986
GAPDH	1:4000 (WB)	Abcam, ab125247
