# Supplementary material for: RANBP2 Activates O-GlcNAcylation through Inducing CEBPα-Dependent OGA Downregulation to Promote Hepatocellular Carcinoma Malignant Phenotypes
Source: Cancers (Basel). 2021 Jul 12;13(14):3475. doi: 10.3390/cancers13143475 (PMC8304650; doi:10.3390/cancers13143475)
Supplement: Supplementary file 1 [file cancers-13-03475-s001.zip › cancers-1259697-suppl-proof/Cells authentication_STR Profiling Hep3B.pdf]

SHANGHAI SAILY BIOTECHNOLOGIES Co., Ltd.

Cell Line Authentication – STR Profiling

Sample Type: Cell Line

Sample from: Central South University

Testing Method: STR Genotyping

Report Time: August 14, 2018

COMPANY STATEMENT

1. THIS REPORT IS ONLY RESPONSIBLE FOR THE SAMPLES ANALYZED.
2. THE TESTING RESULTS AND THE ORGANIZATION NAME WILL NOT BE USED FOR ADVERTISEMENT, COMMERCIAL EXHIBITIONS, COMMERCIAL PERFORMANCE AND OTHER COMMERCIAL ACTIVITIES.
3. OBJECTIONS SHOULD BE RAISED WITHIN FIFTEEN DAYS AFTER THE RECEIPT OF THIS REPORT.
4. THE PAPER REPORT WITH CONTENT ALTERING, ADDING OR WITHOUT THE STAMPED SEAL OF THE COMPANY ARE INVALID.

Testing Company: Shanghai Saily Biotechnologies Co., Ltd.
Address: Room 303, Suite E, 1088 Kang Qiao Dong Rd., Pudong New District, Shanghai201315
Tel: +86-021-60536642
Contact: Yang Xiang
E-mail: info@sailybio.com

Cell Line Authentication – STR Profiling Report

Sample code:

Table 1. Sample Code

Customer's code	Company Code
Hep-3B	20180727-01

Sample Number: 1

Sample Type: Cell line

Testing Type: STR

Sample From:

Central South University

Testing Method:

DNA was extracted by a DNA extraction kit from Axygen, and amplified by 20-STR amplification protocol. The STR locus and sex gene Amelogenin of the DNA were detected on the ABI 3730XL genetic analyzer.

Test Results:

1. Result

Table 2. Matching information on the cell lines

Sample Code	Multi-allele	Cell line matched	Cell Bank	EV	Result
20180727-01	No	Hep-3B	DSMZ	0.94	Basically Matched

- Multi-allele means some STR contain more than two loci.
- The cell typing results of this test are good.

2. Sample Description

20180727-01: The DNA typing of the cell lines found to **basically match** the type of cell lines in a cell line retrieval. DSMZ database shows that the cells called **Hep-3B**, corresponding to the cell number **93**. **No multiple alleles** were found in this cell line.

Note: The cell lines to be tested compared with the cell line STR data collected in ATCC, DSMZ, JCRB, RIKEN database, and the cell lines not included in those cell banks can not be matched.

3. Genotyping Result

Table 3. STR and Amelogenin Genotyping Results of Cell line 20180727-01

Marker	Sample			Cell Bank information		
	Allele1	Allele2	Allele3	Allele1	Allele2	Allele3
D5S818	13	13		13	13	
D13S317	12	14		12	14	
D7S820	8	8		8	10	
D16S539	10	10		10	10	
VWA	17	17		17	17	
TH01	6	7		6	7	
AMEL	X	X		X	X	
TPOX	9	9		9	9	
CSF1PO	8	8		8	8	
D12S391	17	17				
FGA	18	18				
D2S1338	21	25				
D21S11	30	31				
D18S51	20	20				
D8S1179	12	12				
D3S1358	15	15				
D6S1043	12	17				
PENTAE	5	16				
D19S433	12.2	14				
PENTAD	12	14				

Others:

1. Genotyping Strategy and Site Distribution

Attached Table. Experimental Strategy and Sites

	**Strategy 1**	**Strategy 2**	**Strategy 3**	**Strategy 4**	**Strategy 5**	**Strategy 6**
1	D3S1358	D8S1179	D19S433	AMEL	D3S1358	D8S1179
2	VWA	D21S11	TH01	D5S818	VWA	D21S11
3	D7S820	D16S539	D13S317	D12S391	D7S820	D16S539
4	CSF1PO	D2S1338	TPOX	FGA	CSF1PO	D2S1338
5	PENTAE	PENTAD	D18S51		PENTAE	PENTAD
6			D6S1043			

2. DSMZ tools was used to carry on the cell line comparison, which contains 2455 cell lines STR data from ATCC, DSMZ, JCRB, ECACC and RIKEN databases. If the cell is not included in the above cell library, the users need to compare with other databases.

Inspector: Menglu Shen
Reviewer: Yang Bai
Approved: Yiqun Chen
Issue date: August 14, 2018
