# Supplementary material for: RANBP2 Activates O-GlcNAcylation through Inducing CEBPα-Dependent OGA Downregulation to Promote Hepatocellular Carcinoma Malignant Phenotypes
Source: Cancers (Basel). 2021 Jul 12;13(14):3475. doi: 10.3390/cancers13143475 (PMC8304650; doi:10.3390/cancers13143475)
Supplement: Supplementary file 1 [file cancers-13-03475-s001.zip › cancers-1259697-suppl-proof/Cells authentication_STR Profiling HepG2.pdf]

SHANGHAI SAILY BIOTECHNOLOGIES Co., Ltd.

Cell Line Authentication – STR Profiling

Sample Type: Cell Line

Sample from: Central South University

Testing Method: STR Genotyping

Report Time: August 14, 2018

COMPANY STATEMENT

1. THIS REPORT IS ONLY RESPONSIBLE FOR THE SAMPLES ANALYZED.
2. THE TESTING RESULTS AND THE ORGANIZATION NAME WILL NOT BE USED FOR ADVERTISEMENT, COMMERCIAL EXHIBITIONS, COMMERCIAL PERFORMANCE AND OTHER COMMERCIAL ACTIVITIES.
3. OBJECTIONS SHOULD BE RAISED WITHIN FIFTEEN DAYS AFTER THE RECEIPT OF THIS REPORT.
4. THE PAPER REPORT WITH CONTENT ALTERING, ADDING OR WITHOUT THE STAMPED SEAL OF THE COMPANY ARE INVALID.

Testing Company: Shanghai Saily Biotechnologies Co., Ltd.
Address: Room 303, Suite E, 1088 Kang Qiao Dong Rd., Pudong New District, Shanghai201315
Tel: +86-021-60536642
Contact: Yang Xiang
E-mail: info@sailybio.com

Cell Line Authentication – STR Profiling Report

Sample code:

Table 1. Sample Code

Customer’s code	Company Code
Hep-G2	20180727-02

Sample Number: 1

Sample Type: Cell line

Testing Type: STR

Sample From:

Central South University

Testing Method：

DNA was extracted by a DNA extraction kit from Axygen, and amplified by 20-STR amplification protocol. The STR locus and sex gene Amelogenin of the DNA were detected on the ABI 3730XL genetic analyzer.

Test Results:

1. Result

Table 2. Matching information on the cell lines

Sample Code	Multi-allele	Cell line matched	Cell Bank	EV	Result
20180727-02	No	Hep-G2	DSMZ	1.00	Perfectly Matched

- Multi-allele means some STR contain more than two loci.
- The cell typing results of this test are good.

2. Sample Description

20180727-02: The DNA typing of the cell lines found to **perfectly match** the type of cell lines in a cell line retrieval. DSMZ database shows that the cells called **GS-HepG2**, corresponding to the cell number **RCB-1681**. **No multiple alleles** were found in this cell line.

Note: The cell lines to be tested compared with the cell line STR data collected in ATCC, DSMZ, JCRB, RIKEN database, and the cell lines not included in those cell banks can not be matched.

3. Genotyping Result

Table 3. STR and Amelogenin Genotyping Results of Cell line 20180727-02

Marker	Sample			Cell Bank information		
	Allele1	Allele2	Allele3	Allele1	Allele2	Allele3
D5S818	11	12		11	12	
D13S317	9	13		9	13	
D7S820	10	10		10	10	
D16S539	12	12		12	12	
VWA	17	17		17	17	
TH01	9	9		9	9	
AMEL	X	Y		X	Y	
TPOX	8	9		8	9	
CSF1PO	10	11		10	11	
D12S391	21	25				
FGA	22	25				
D2S1338	19	20				
D21S11	13	14				
D18S51	15	16				
D8S1179	15	16				
D3S1358	13	13				
D6S1043	15	20				
PENTAE	15.2	15.2				
D19S433	9	13				
PENTAD	11	12				

Others:

1. Genotyping Strategy and Site Distribution

Attached Table. Experimental Strategy and Sites

	**Strategy 1**	**Strategy 2**	**Strategy 3**	**Strategy 4**	**Strategy 5**	**Strategy 6**
1	TH01	AMEL	TPOX	D3S1358	PENTAE	D19S433
2	D12S391	D5S818	VWA	D13S317		PENTAD
3	D7S820	D2S1338	D8S1179	D6S1043		
4	CSF1PO	D18S51		D16S539		
5	FGA					

2. DSMZ tools was used to carry on the cell line comparison, which contains 2455 cell lines STR data from ATCC, DSMZ, JCRB, ECACC and RIKEN databases. If the cell is not included in the above cell library, the users need to compare with other databases.

Inspector: Menglu Shen
Reviewer: Yang Bai
Approved: Yiqun Chen
Issue date: August 14, 2018
