# Supplementary material for: RANBP2 Activates O-GlcNAcylation through Inducing CEBPα-Dependent OGA Downregulation to Promote Hepatocellular Carcinoma Malignant Phenotypes
Source: Cancers (Basel). 2021 Jul 12;13(14):3475. doi: 10.3390/cancers13143475 (PMC8304650; doi:10.3390/cancers13143475)
Supplement: Supplementary file 1 [file cancers-13-03475-s001.zip › cancers-1259697-suppl-proof/Original Data_Revision/1.qPCR data_Figure 4E_CHIP assay.pdf]

qPCR data_ Figure 4E_ CHIP assay

Cell Lines	Group	Sample Name	Cт	ΔCт	ΔΔCт	RQ	RQ mean	Expression (mean) in CHIP group
**Hep3B**	A-IgG	A	30.6799	12.1598	0	1	1.1099	1.1099
	A-IgG	A	30.7453	11.8987	0.2611	1.1984		
	A-IgG	A	30.8037	11.9819	0.1779	1.1312		
	A-CHIP	B	27.1537	8.6336	3.5262	11.521	13.7853	11.0138
	A-CHIP	B	27.0148	8.1682	3.9916	15.9071		
	A-CHIP	B	27.1817	8.3599	3.7999	13.9278		
	A-Input	C	26.9173	8.3972	3.7626	13.5724	17.2543	
	A-Input	C	26.735	7.8884	4.2714	19.3117		
	A-Input	C	26.7429	7.9211	4.2387	18.8789		
	A-GAPDH	D-internal	18.5201					
	A-GAPDH	D-internal	18.8466					
	A-GAPDH	D-internal	18.8218					
	B-IgG	E	31.1508	12.3879	0.3248	1.2525	1.1518	1.1518
	B-IgG	E	31.2261	12.7127	0	1		
	B-IgG	E	31.2262	12.4461	0.2666	1.203		
	B-CHIP	F	27.1207	8.3578	4.3549	20.4624	19.0538	16.937
	B-CHIP	F	27.2334	8.72	3.9927	15.9192		
	B-CHIP	F	27.1157	8.3356	4.3771	20.7797		
	B-Input	G	26.9415	8.1786	4.5341	23.1686	21.435	
	B-Input	G	27.1123	8.5989	4.1138	17.3132		
	B-Input	G	26.9185	8.1384	4.5743	23.8233		
	B-GAPDH	H-internal	18.7629					
	B-GAPDH	H-internal	18.5134					
	B-GAPDH	H-internal	18.7801					
	C-IgG	I	30.4259	12.0845	0.1313	1.0953	1.0575	1.0575
	C-IgG	I	30.75	12.2158	0	1		
	C-IgG	I	30.6251	12.1083	0.1075	1.0774		
	C-CHIP	J	27.6074	9.266	2.9498	7.7264	8.846	5.6096
	C-CHIP	J	27.4787	8.9445	3.2713	9.6552		
	C-CHIP	J	27.5378	9.021	3.1948	9.1565		
	C-Input	K	26.8827	8.5413	3.6745	12.7683	13.9497	
	C-Input	K	26.92	8.3858	3.83	14.2215		
	C-Input	K	26.8393	8.3225	3.8933	14.8594		
	C-GAPDH	L-internal	18.3414					
	C-GAPDH	L-internal	18.5342					
	C-GAPDH	L-internal	18.5168					
HepG2	D-IgG	M	31.2316	12.0144	0.2989	1.2302	1.1869	1.1869
	D-IgG	M	31.36	12.3133	0	1		
	D-IgG	M	31.1468	11.9013	0.412	1.3305		
	D-CHIP	N	27.2834	8.0662	4.1496	17.7482	17.1213	14.605
	D-CHIP	N	27.3122	8.2655	3.9503	15.4582		
	D-CHIP	N	27.2788	8.0333	4.1825	18.1576		
	D-Input	O	26.973	7.7558	4.46	22.0087	20.0712	
	D-Input	O	27.106	8.0593	4.1565	17.8333		
	D-Input	O	27.1128	7.8673	4.3485	20.3718		
	D-GAPDH	P-internal	19.2172					
	D-GAPDH	P-internal	19.0467					
	D-GAPDH	P-internal	19.2455					
	E-IgG	Q	31.288	11.8444	0.0597	1.0422	1.0175	1.0175
	E-IgG	Q	31.3014	11.8892	0.0149	1.0104		
	E-IgG	Q	31.1775	11.9041	0	1		

E-CHIP	R	27.1593	7.7157	4.5001	22.629	22.2378	21.0262
E-CHIP	R	27.1546	7.7424	4.4734	22.214		
E-CHIP	R	27.0383	7.7649	4.4509	21.8703		
E-Input	S	26.9133	7.4697	4.7461	26.836	23.5192	
E-Input	S	27.1257	7.7135	4.5023	22.6635		
E-Input	S	27.0929	7.8195	4.3963	21.0581		
E-GAPDH	T-internal	19.4436					
E-GAPDH	T-internal	19.4122					
E-GAPDH	T-internal	19.2734					
F-IgG	U	31.0249	11.9289	0	1	1.0754	1.0754
F-IgG	U	30.9473	11.6532	0.2757	1.2106		
F-IgG	U	31.0402	11.9064	0.0225	1.0157		
F-CHIP	V	28.1146	9.0186	2.9103	7.5177	8.4832	4.9193
F-CHIP	V	27.9856	8.6915	3.2374	9.4309		
F-CHIP	V	27.9751	8.8413	3.0876	8.5008		
F-Input	W	27.1378	8.0418	3.8871	14.7956	14.6289	
F-Input	W	27.3078	8.0137	3.9152	15.0866		
F-Input	W	27.2549	8.1211	3.8078	14.0043		
F-GAPDH	X	19.096					
F-GAPDH	X	19.2941					
F-GAPDH	X	19.1338					
