# Supplementary material for: RANBP2 Activates O-GlcNAcylation through Inducing CEBPα-Dependent OGA Downregulation to Promote Hepatocellular Carcinoma Malignant Phenotypes
Source: Cancers (Basel). 2021 Jul 12;13(14):3475. doi: 10.3390/cancers13143475 (PMC8304650; doi:10.3390/cancers13143475)
Supplement: Supplementary file 1 [file cancers-13-03475-s001.zip › cancers-1259697-suppl-proof/Original Data_Revision/2.qPCR data_Supplemental Figure 3B_OGT & OGA mRNA expression.pdf]

qPCR data_Supplemental Figure 3B_OGT & OGA mRNA expression

Hep3B OGT

Well	Sample Name	CT	RQ	RQ Mean	ΔCT	_-ΔΔCT
A1	A	27.094	1.0955	1.0872	8.4479	0.1316
A2	A	27.2509	1		8.5795	0
A3	A	27.2658	1.1661		8.3578	0.2217
A4	A-GAPDH	18.6461				
A5	A-GAPDH	18.6714				
A6	A-GAPDH	18.908				
B1	B	27.1773	0.9318	0.9579	8.6814	-0.1019
B2	B	27.1879	0.967		8.6279	-0.0484
B3	B	27.0309	0.9748		8.6163	-0.0368
B4	B-GAPDH	18.4959				
B5	B-GAPDH	18.56				
B6	B-GAPDH	18.4146				

HepG2 OGT

Well	Sample Name	CT	RQ	RQ Mean	ΔCT	_-ΔΔCT
A1	A	27.4706	1.0795	1.0324	8.9968	0.1104
A2	A	27.4536	1		9.1072	0
A3	A	27.5249	1.0176		9.0821	0.0251
A4	A-GAPDH	18.4738				
A5	A-GAPDH	18.3464				
A6	A-GAPDH	18.4428				
B1	B	28.2783	0.9697	0.9991	9.1516	-0.0444
B2	B	27.9515	1.0222		9.0755	0.0317
B3	B	27.9587	1.0053		9.0996	0.0076
B4	B-GAPDH	19.1267				
B5	B-GAPDH	18.876				
B6	B-GAPDH	18.8591				

Hep3B OGA

Well	Sample Name	Cт	RQ	RQ Mean	ΔCт	_-ΔΔCт
A1	A	27.3073	1	1.0542	8.465	0
A2	A	27.3135	1.0567		8.3855	0.0795
A3	A	27.1049	1.106		8.3197	0.1453
A4	A-GAPDH	18.8423				
A5	A-GAPDH	18.928				
A6	A-GAPDH	18.7852				
B1	B	25.5998	3.2564	3.136	6.7617	1.7033
B2	B	26.0018	2.9982		6.8809	1.5841
B3	B	25.6629	3.1532		6.8082	1.6568
B4	B-GAPDH	18.8381				
B5	B-GAPDH	19.1209				
B6	B-GAPDH	18.8547				

HepG2 OGA

Well	Sample Name	Cт	RQ	RQ Mean	ΔCт	_-ΔΔCт
A1	A	27.435	1.0575	1.0569	8.8677	0.0806
A2	A	27.4569	1		8.9483	0
A3	A	27.3525	1.1132		8.7936	0.1547
A4	A-GAPDH	18.5673				
A5	A-GAPDH	18.5086				
A6	A-GAPDH	18.5589				
B1	B	26.4244	2.5489	2.6337	7.5984	1.3499
B2	B	26.4109	2.7313		7.4987	1.4496
B3	B	26.6926	2.621		7.5582	1.3901
B4	B-GAPDH	18.826				
B5	B-GAPDH	18.9122				
B6	B-GAPDH	19.1344				
