# Supplementary material for: RANBP2 Activates O-GlcNAcylation through Inducing CEBPα-Dependent OGA Downregulation to Promote Hepatocellular Carcinoma Malignant Phenotypes
Source: Cancers (Basel). 2021 Jul 12;13(14):3475. doi: 10.3390/cancers13143475 (PMC8304650; doi:10.3390/cancers13143475)
Supplement: Supplementary file 1 [file cancers-13-03475-s001.zip › cancers-1259697-suppl-proof/Original Data_Revision/3.qPCR data_Supplemental Figure 3C_OGA mRNA stability assay.pdf]

qPCR data_Supplemental Figure 3C_OGA mRNA stability assay

Hep3B cell

Well	Sample Name	C_T	RQ	RQ Mean	ΔC_T	$\Delta\Delta C_T$	Time elapsed	Annotation
A1	A	27.385	1.0072	1.0037	-0.0103	0.0103	**A-0h**	A = NC group
A2	A	27.3454	0.9659		0.0501	-0.0501		B = sh-RANBP2 group
A3	A	27.2941	1.0382		-0.0541	0.0541		
A4	A-18S	18.7638	1.0133	0.9834	-0.019	0.019		
A5	A-18S	18.9236	0.9286		0.1068	-0.1068		
A6	A-18S	18.7764	1.0083		-0.0119	0.0119		
A7	A-β-Actin	17.4305	1.0185	1.0059	-0.0264	0.0264		
A8	A-β-Actin	17.4405	1.0332		-0.0471	0.0471		
A9	A-β-Actin	17.5577	0.9659		0.05	-0.05		
B1	B	27.3446	1.0358	0.8958	-0.0507	0.0507	**A-2h**	
B2	B	27.6315	0.7921		0.3362	-0.3362		
B3	B	27.5666	0.8595		0.2184	-0.2184		
B4	B-18S	18.8926	0.9267	0.9534	0.1098	-0.1098		
B5	B-18S	18.8267	0.9932		0.0099	-0.0099		
B6	B-18S	18.8773	0.9402		0.089	-0.089		
B7	B-β-Actin	17.4123	1.0314	0.9937	-0.0446	0.0446		
B8	B-β-Actin	17.4744	1.0092		-0.0132	0.0132		
B9	B-β-Actin	17.5963	0.9404		0.0886	-0.0886		
C1	C	27.4606	0.9557	0.8329	0.0653	-0.0653	**A-4h**	
C2	C	27.7051	0.7527		0.4098	-0.4098		
C3	C	27.6877	0.7903		0.3395	-0.3395		
C4	C-18S	18.9328	0.9013	0.965	0.15	-0.15		
C5	C-18S	18.83	0.9909		0.0132	-0.0132		
C6	C-18S	18.7842	1.0028		-0.0041	0.0041		
C7	C-β-Actin	17.6145	0.8965	0.9368	0.1576	-0.1576		
C8	C-β-Actin	17.5594	0.9515		0.0718	-0.0718		
C9	C-β-Actin	17.5629	0.9625		0.0552	-0.0552		
D1	D	27.8694	0.7199	0.7456	0.4741	-0.4741	**A-6h**	
D2	D	27.6679	0.7724		0.3726	-0.3726		
D3	D	27.7739	0.7445		0.4257	-0.4257		
D4	D-18S	18.8634	0.9457	0.9168	0.0806	-0.0806		
D5	D-18S	18.9572	0.9073		0.1404	-0.1404		
D6	D-18S	18.9442	0.8976		0.1559	-0.1559		
D7	D-β-Actin	17.6332	0.885	0.886	0.1763	-0.1763		
D8	D-β-Actin	17.6405	0.8994		0.1529	-0.1529		
D9	D-β-Actin	17.7026	0.8736		0.1949	-0.1949		
E1	E	27.9082	0.7008	0.6977	0.5129	-0.5129	**A-8h**	
E2	E	27.7796	0.7148		0.4843	-0.4843		
E3	E	27.91	0.6775		0.5618	-0.5618		
E4	E-18S	18.9349	0.8999	0.9541	0.1521	-0.1521		
E5	E-18S	18.8024	1.01		-0.0144	0.0144		
E6	E-18S	18.8586	0.9524		0.0703	-0.0703		
E7	E-β-Actin	17.7003	0.8448	0.8527	0.2434	-0.2434		
E8	E-β-Actin	17.6957	0.8657		0.2081	-0.2081		
E9	E-β-Actin	17.7461	0.8477		0.2384	-0.2384		
F1	F	27.9934	0.6606	0.6389	0.5981	-0.5981	**A-12h**	
F2	F	27.9027	0.6564		0.6074	-0.6074		

F3	F	28.0858	0.5997		0.7376	-0.7376	
F4	F-18S	18.906	0.9181	0.9117	0.1232	-0.1232	
F5	F-18S	18.9	0.944		0.0832	-0.0832	
F6	F-18S	18.9843	0.873		0.196	-0.196	
F7	F-β-Actin	17.8532	0.7598	0.8118	0.3963	-0.3963	
F8	F-β-Actin	17.7137	0.8549		0.2261	-0.2261	
F9	F-β-Actin	17.793	0.8206		0.2853	-0.2853	
G1	G	27.4384	0.9706	0.9781	0.0431	-0.0431	**B-0h**
G2	G	27.2655	1.0209		-0.0298	0.0298	
G3	G	27.4331	0.9428		0.0849	-0.0849	
G4	G-18S	18.8177	0.9761	0.9881	0.0349	-0.0349	
G5	G-18S	18.8593	0.971		0.0425	-0.0425	
G6	G-18S	18.7637	1.0172		-0.0246	0.0246	
G7	G-β-Actin	17.571	0.924	0.987	0.1141	-0.1141	
G8	G-β-Actin	17.4815	1.0042		-0.0061	0.0061	
G9	G-β-Actin	17.4613	1.0327		-0.0464	0.0464	
H1	H	27.6362	0.8462	0.8411	0.2409	-0.2409	**B-2h**
H2	H	27.6292	0.7934		0.3339	-0.3339	
H3	H	27.5265	0.8837		0.1783	-0.1783	
H4	H-18S	18.9206	0.9089	0.9611	0.1378	-0.1378	
H5	H-18S	18.7165	1.072		-0.1003	0.1003	
H6	H-18S	18.9367	0.9023		0.1484	-0.1484	
H7	H-β-Actin	17.6188	0.8938	1.0025	0.1619	-0.1619	
H8	H-β-Actin	17.4135	1.0527		-0.0741	0.0741	
H9	H-β-Actin	17.4225	1.0608		-0.0852	0.0852	
I1	I	27.7956	0.7577	0.704	0.4003	-0.4003	**B-4h**
I2	I	27.8246	0.6929		0.5293	-0.5293	
I3	I	27.9448	0.6613		0.5966	-0.5966	
I4	I-18S	18.9285	0.9039	0.9562	0.1457	-0.1457	
I5	I-18S	18.8297	0.9911		0.0129	-0.0129	
I6	I-18S	18.8271	0.9735		0.0388	-0.0388	
I7	I-β-Actin	17.6191	0.8937	0.9166	0.1622	-0.1622	
I8	I-β-Actin	17.6453	0.8965		0.1577	-0.1577	
I9	I-β-Actin	17.5671	0.9597		0.0594	-0.0594	
J1	J	28.1278	0.6019	0.6035	0.7325	-0.7325	**B-6h**
J2	J	28.0157	0.6069		0.7204	-0.7204	
J3	J	28.081	0.6017		0.7328	-0.7328	
J4	J-18S	19.0321	0.8413	0.9074	0.2493	-0.2493	
J5	J-18S	18.9354	0.9211		0.1186	-0.1186	
J6	J-18S	18.8475	0.9598		0.0592	-0.0592	
J7	J-β-Actin	17.623	0.8912	0.8784	0.1661	-0.1661	
J8	J-β-Actin	17.7237	0.849		0.2361	-0.2361	
J9	J-β-Actin	17.668	0.8948		0.1603	-0.1603	
K1	K	28.3521	0.5152	0.5608	0.9568	-0.9568	**B-8h**
K2	K	28.1788	0.5421		0.8835	-0.8835	
K3	K	28.0256	0.6253		0.6774	-0.6774	
K4	K-18S	18.9516	0.8896	0.9364	0.1688	-0.1688	
K5	K-18S	18.8457	0.9802		0.0289	-0.0289	
K6	K-18S	18.8786	0.9393		0.0903	-0.0903	
K7	K-β-Actin	17.8512	0.7609	0.8126	0.3943	-0.3943	
K8	K-β-Actin	17.7666	0.8242		0.279	-0.279	
K9	K-β-Actin	17.7375	0.8528		0.2298	-0.2298	

L1	L	28.1986	0.573	0.5244	0.8033	-0.8033	**B-12h**
L2	L	28.3513	0.481		1.056	-1.056	
L3	L	28.294	0.5191		0.9458	-0.9458	
L4	L-18S	19.0401	0.8367	0.9015	0.2573	-0.2573	
L5	L-18S	18.8618	0.9693		0.045	-0.045	
L6	L-18S	18.9425	0.8986		0.1542	-0.1542	
L7	L-β-Actin	17.764	0.8083	0.7897	0.3071	-0.3071	
L8	L-β-Actin	17.8879	0.7577		0.4003	-0.4003	
L9	L-β-Actin	17.8241	0.8031		0.3164	-0.3164	

HepG2 cell

Well	Sample Na	Cт	RQ	RQ Mean	ΔCт	ΔΔCт	Time elapsed	Annotation
A1	A	26.4418	1	1	0	0	**A-0h**	A = NC group
A2	A	26.2541	1		0	0		B = sh-RANBP2 group
A3	A	26.424	1		0	0		
A4	A-18S	18.1638	1	1	0	0		
A5	A-18S	18.2227	1		0	0		
A6	A-18S	18.3667	1		0	0		
A7	A-β-Actin	17.2541	1	1	0	0		
A8	A-β-Actin	17.1049	1		0	0		
A9	A-β-Actin	17.226	1		0	0		
B1	B	26.7331	0.8172	0.8167	0.2913	-0.2913	**A-2h**	
B2	B	26.5867	0.7941		0.3326	-0.3326		
B3	B	26.6776	0.8388		0.2536	-0.2536		
B4	B-18S	18.2918	0.9151	0.9986	0.128	-0.128		
B5	B-18S	18.2013	1.0149		-0.0214	0.0214		
B6	B-18S	18.275	1.0656		-0.0917	0.0917		
B7	B-β-Actin	17.1665	1.0626	0.9564	-0.0876	0.0876		
B8	B-β-Actin	17.3026	0.8719		0.1977	-0.1977		
B9	B-β-Actin	17.3233	0.9348		0.0973	-0.0973		
C1	C	27.071	0.6465	0.6495	0.6292	-0.6292	**A-4h**	
C2	C	27.0228	0.5869		0.7687	-0.7687		
C3	C	26.9081	0.7149		0.4841	-0.4841		
C4	C-18S	18.2267	0.9573	0.988	0.0629	-0.0629		
C5	C-18S	18.1888	1.0238		-0.0339	0.0339		
C6	C-18S	18.3916	0.9829		0.0249	-0.0249		
C7	C-β-Actin	17.3458	0.9384	0.9337	0.0917	-0.0917		
C8	C-β-Actin	17.2708	0.8914		0.1659	-0.1659		
C9	C-β-Actin	17.2678	0.9714		0.0418	-0.0418		
D1	D	27.3238	0.5426	0.5723	0.882	-0.882	**A-6h**	
D2	D	27.1435	0.5398		0.8894	-0.8894		
D3	D	27.0801	0.6346		0.6561	-0.6561		
D4	D-18S	18.3591	0.8734	0.9051	0.1953	-0.1953		
D5	D-18S	18.4326	0.8646		0.2099	-0.2099		
D6	D-18S	18.3999	0.9773		0.0332	-0.0332		
D7	D-β-Actin	17.3426	0.9405	0.8735	0.0885	-0.0885		
D8	D-β-Actin	17.4035	0.813		0.2986	-0.2986		
D9	D-β-Actin	17.432	0.8669		0.206	-0.206		
E1	E	27.4976	0.481	0.4683	1.0558	-1.0558	**A-8h**	

E2	E	27.4528	0.4357		1.1987	-1.1987	
E3	E	27.4582	0.4883		1.0342	-1.0342	
E4	E-18S	18.3433	0.883	0.9213	0.1795	-0.1795	
E5	E-18S	18.3777	0.8981		0.155	-0.155	
E6	E-18S	18.3918	0.9828		0.0251	-0.0251	
E7	E-β-Actin	17.3922	0.9087	0.8478	0.1381	-0.1381	
E8	E-β-Actin	17.4108	0.8089		0.3059	-0.3059	
E9	E-β-Actin	17.5021	0.8258		0.2761	-0.2761	
F1	F	27.654	0.4316	0.4025	1.2122	-1.2122	**A-12h**
F2	F	27.7632	0.3513		1.5091	-1.5091	
F3	F	27.6598	0.4246		1.2358	-1.2358	
F4	F-18S	18.2553	0.9385	0.9135	0.0915	-0.0915	
F5	F-18S	18.4769	0.8385		0.2542	-0.2542	
F6	F-18S	18.4202	0.9636		0.0535	-0.0535	
F7	F-β-Actin	17.4454	0.8758	0.8183	0.1913	-0.1913	
F8	F-β-Actin	17.5132	0.7535		0.4083	-0.4083	
F9	F-β-Actin	17.5024	0.8256		0.2764	-0.2764	
G1	G	26.342	1.0716	1.0224	-0.0998	0.0998	**B-0h**
G2	G	26.3748	0.9197		0.1207	-0.1207	
G3	G	26.3187	1.0757		-0.1053	0.1053	
G4	G-18S	18.1942	0.9791	0.9954	0.0304	-0.0304	
G5	G-18S	18.339	0.9226		0.1163	-0.1163	
G6	G-18S	18.2497	1.0845		-0.117	0.117	
G7	G-β-Actin	17.1531	1.0725	1.0332	-0.101	0.101	
G8	G-β-Actin	17.1157	0.9925		0.0108	-0.0108	
G9	G-β-Actin	17.1771	1.0345		-0.0489	0.0489	
H1	H	26.4971	0.8563	0.8294	0.1551	-0.1551	**B-2h**
H2	H	26.5983	0.7997		0.2235	-0.2235	
H3	H	26.5025	0.8321		0.1838	-0.1838	
H4	H-18S	18.298	0.9112	0.9853	0.1342	-0.1342	
H5	H-18S	18.3318	0.9272		0.1091	-0.1091	
H6	H-18S	18.2062	1.1177		-0.1605	0.1605	
H7	H-β-Actin	17.2929	0.9735	0.977	0.0388	-0.0388	
H8	H-β-Actin	17.2841	0.8832		0.1792	-0.1792	
H9	H-β-Actin	17.1227	1.0742		-0.1033	0.1033	
I1	I	26.8035	0.6303	0.6156	0.4615	-0.4615	**B-4h**
I2	I	26.8519	0.6206		0.4771	-0.4771	
I3	I	26.8166	0.6078		0.4979	-0.4979	
I4	I-18S	18.3219	0.8962	0.9594	0.1581	-0.1581	
I5	I-18S	18.234	0.9922		0.0113	-0.0113	
I6	I-18S	18.3815	0.9898		0.0148	-0.0148	
I7	I-β-Actin	17.3449	0.939	0.919	0.0908	-0.0908	
I8	I-β-Actin	17.3295	0.8558		0.2246	-0.2246	
I9	I-β-Actin	17.2818	0.9621		0.0558	-0.0558	
J1	J	26.8369	0.6096	0.6204	0.4949	-0.4949	**B-6h**
J2	J	26.7992	0.6541		0.4244	-0.4244	
J3	J	26.8324	0.5977		0.5146	-0.5146	
J4	J-18S	18.4365	0.8278	0.9532	0.2727	-0.2727	
J5	J-18S	18.2763	0.9635		0.0536	-0.0536	
J6	J-18S	18.2714	1.0683		-0.0953	0.0953	
J7	J-β-Actin	17.4316	0.8842	0.8737	0.1775	-0.1775	
J8	J-β-Actin	17.3477	0.8451		0.2428	-0.2428	

J9	J-β-Actin	17.3913	0.8917		0.1653	-0.1653	
K1	K	26.9894	0.5234	0.5029	0.6474	-0.6474	**B-8h**
K2	K	27.0292	0.5244		0.6544	-0.6544	
K3	K	27.0935	0.4608		0.7748	-0.7748	
K4	K-18S	18.4126	0.8416	0.9146	0.2488	-0.2488	
K5	K-18S	18.4065	0.8804		0.1838	-0.1838	
K6	K-18S	18.3356	1.0218		-0.0311	0.0311	
K7	K-β-Actin	17.4914	0.8483	0.8567	0.2373	-0.2373	
K8	K-β-Actin	17.3664	0.8342		0.2615	-0.2615	
K9	K-β-Actin	17.3983	0.8874		0.1723	-0.1723	
L1	L	27.1113	0.4633	0.418	0.7693	-0.7693	**B-12h**
L2	L	27.2782	0.4052		0.9034	-0.9034	
L3	L	27.2716	0.3856		0.9529	-0.9529	
L4	L-18S	18.377	0.8626	0.9183	0.2132	-0.2132	
L5	L-18S	18.4186	0.873		0.1959	-0.1959	
L6	L-18S	18.3394	1.0191		-0.0273	0.0273	
L7	L-β-Actin	17.4713	0.8602	0.8398	0.2172	-0.2172	
L8	L-β-Actin	17.5156	0.7523		0.4107	-0.4107	
L9	L-β-Actin	17.367	0.9069		0.141	-0.141	
