# Supplementary material for: RANBP2 Activates O-GlcNAcylation through Inducing CEBPα-Dependent OGA Downregulation to Promote Hepatocellular Carcinoma Malignant Phenotypes
Source: Cancers (Basel). 2021 Jul 12;13(14):3475. doi: 10.3390/cancers13143475 (PMC8304650; doi:10.3390/cancers13143475)
Supplement: Supplementary file 1 [file cancers-13-03475-s001.zip › cancers-1259697-suppl-proof/Original Data_Revision/4.Original WB Figures_Revision.pptx]

## Slide 1
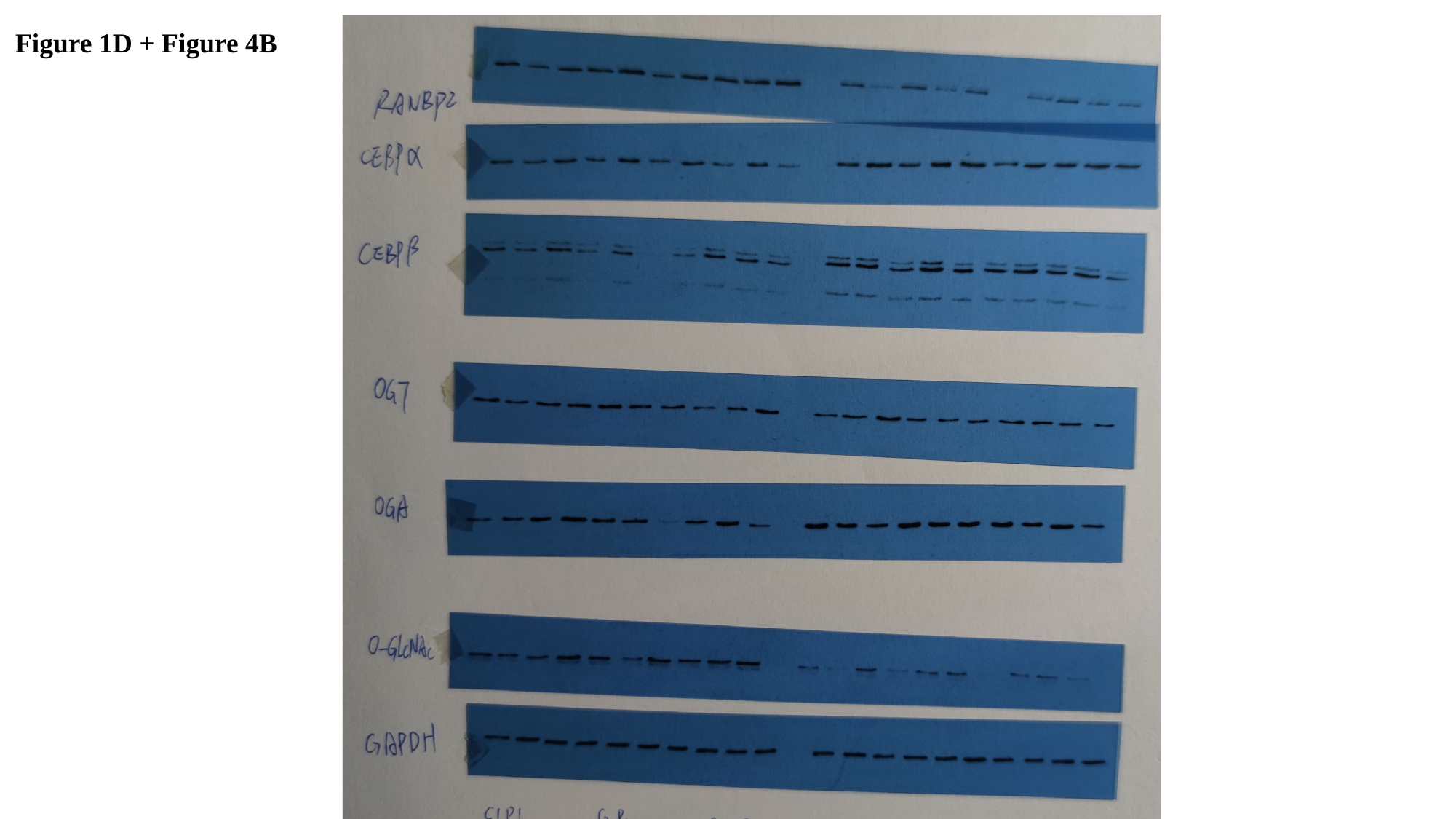

Figure 1D + Figure 4B

## Slide 2
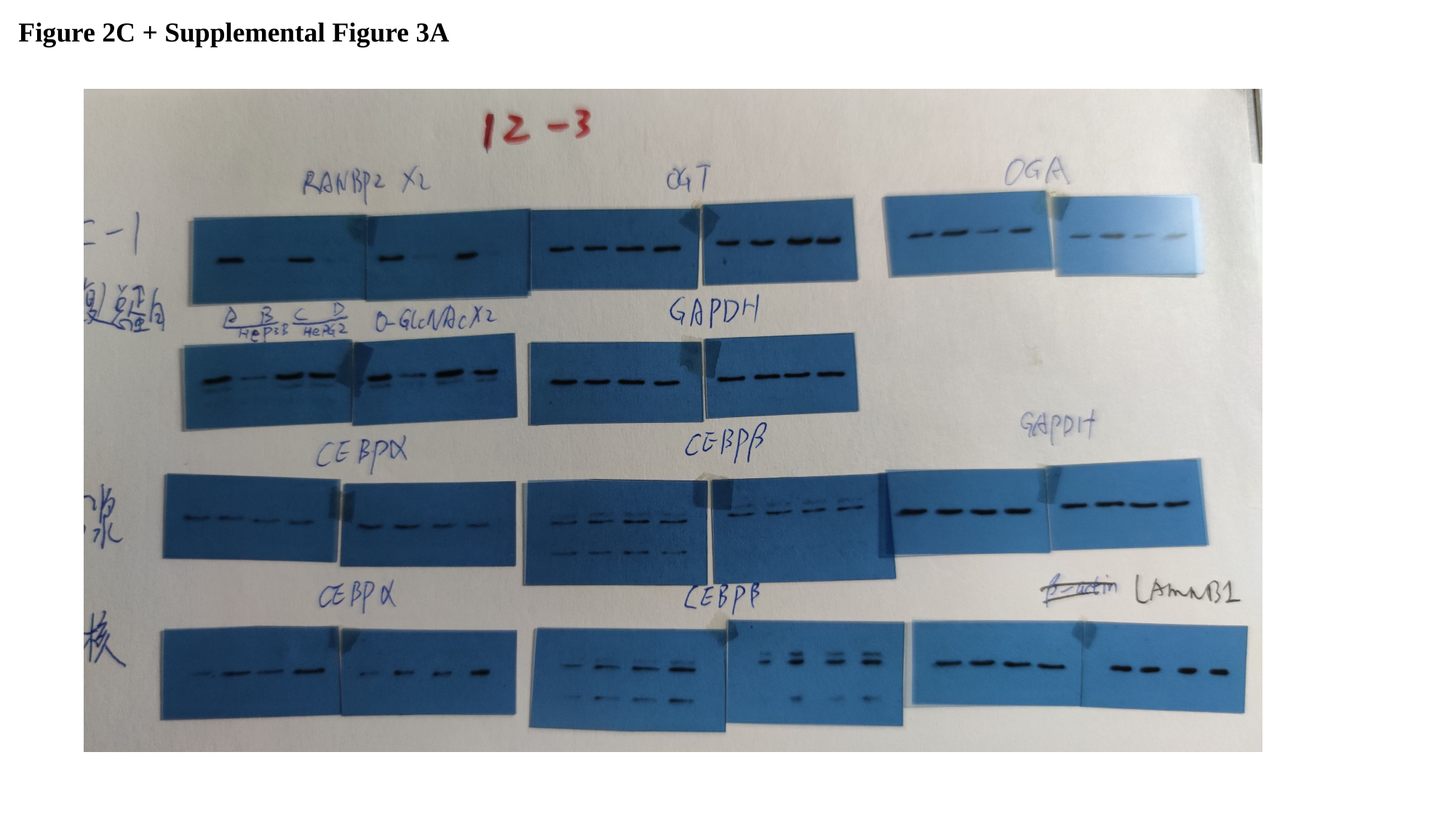

Figure 2C + Supplemental Figure 3A

## Slide 3
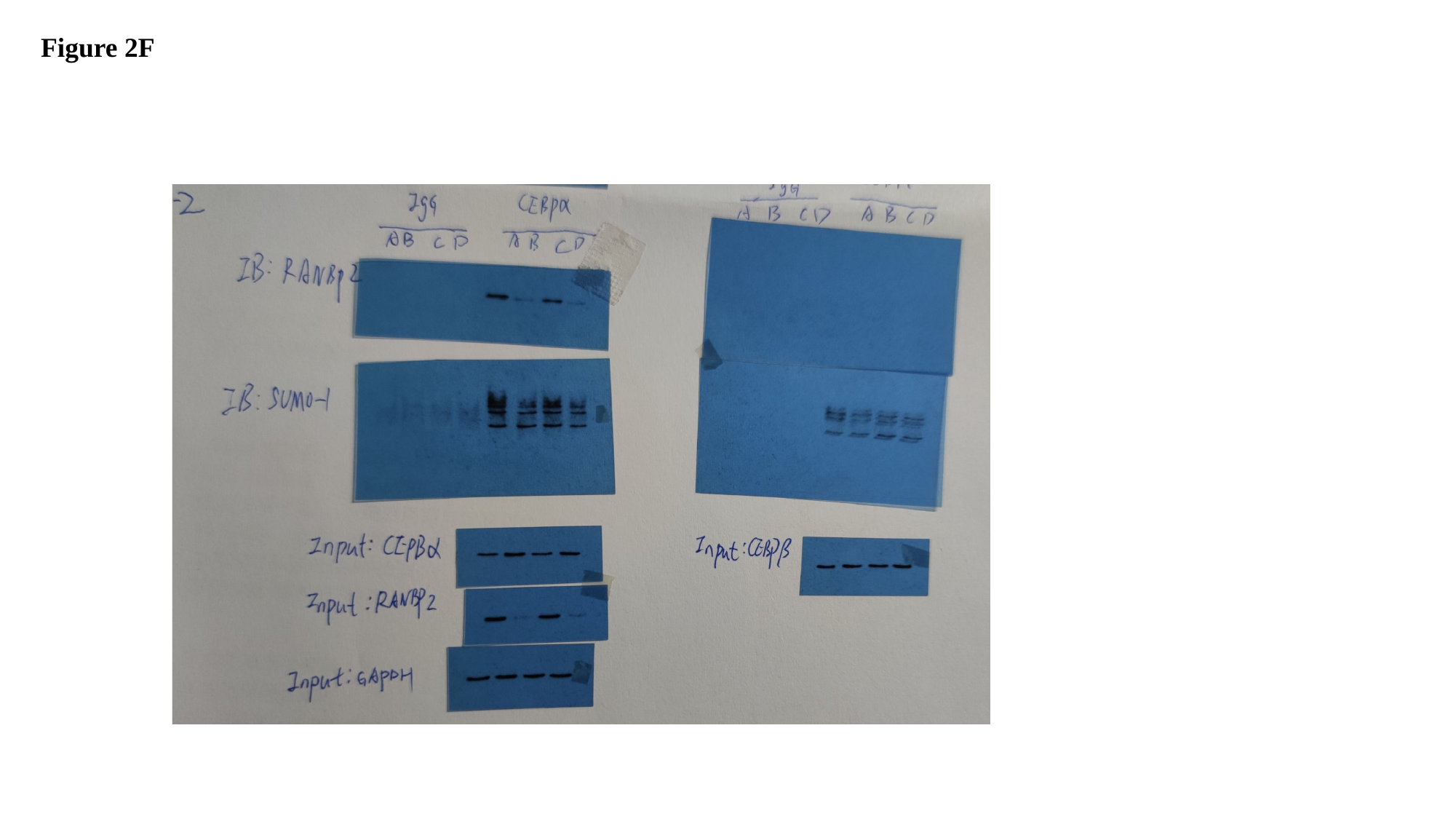

Figure 2F

## Slide 4
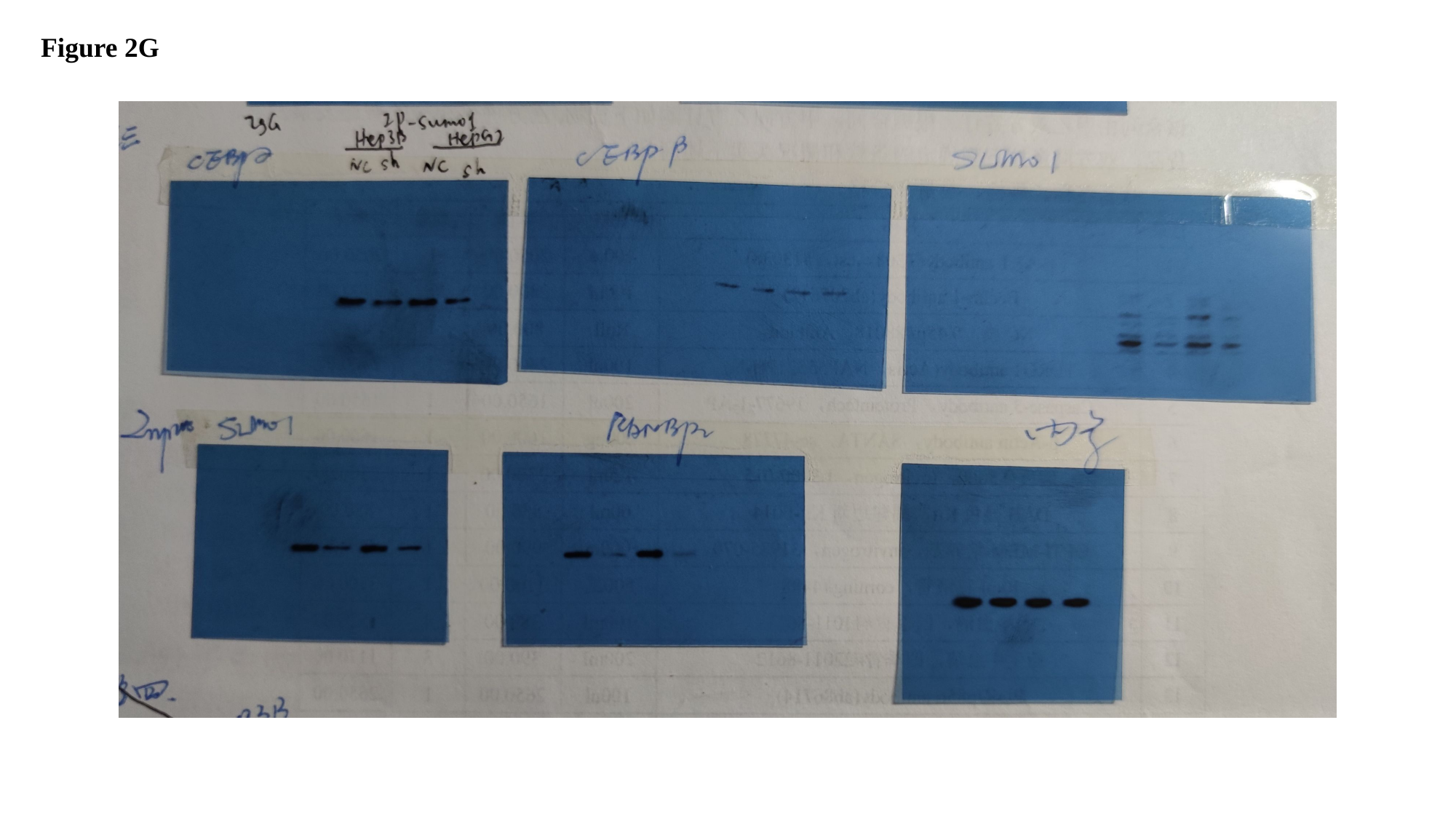

Figure 2G

## Slide 5
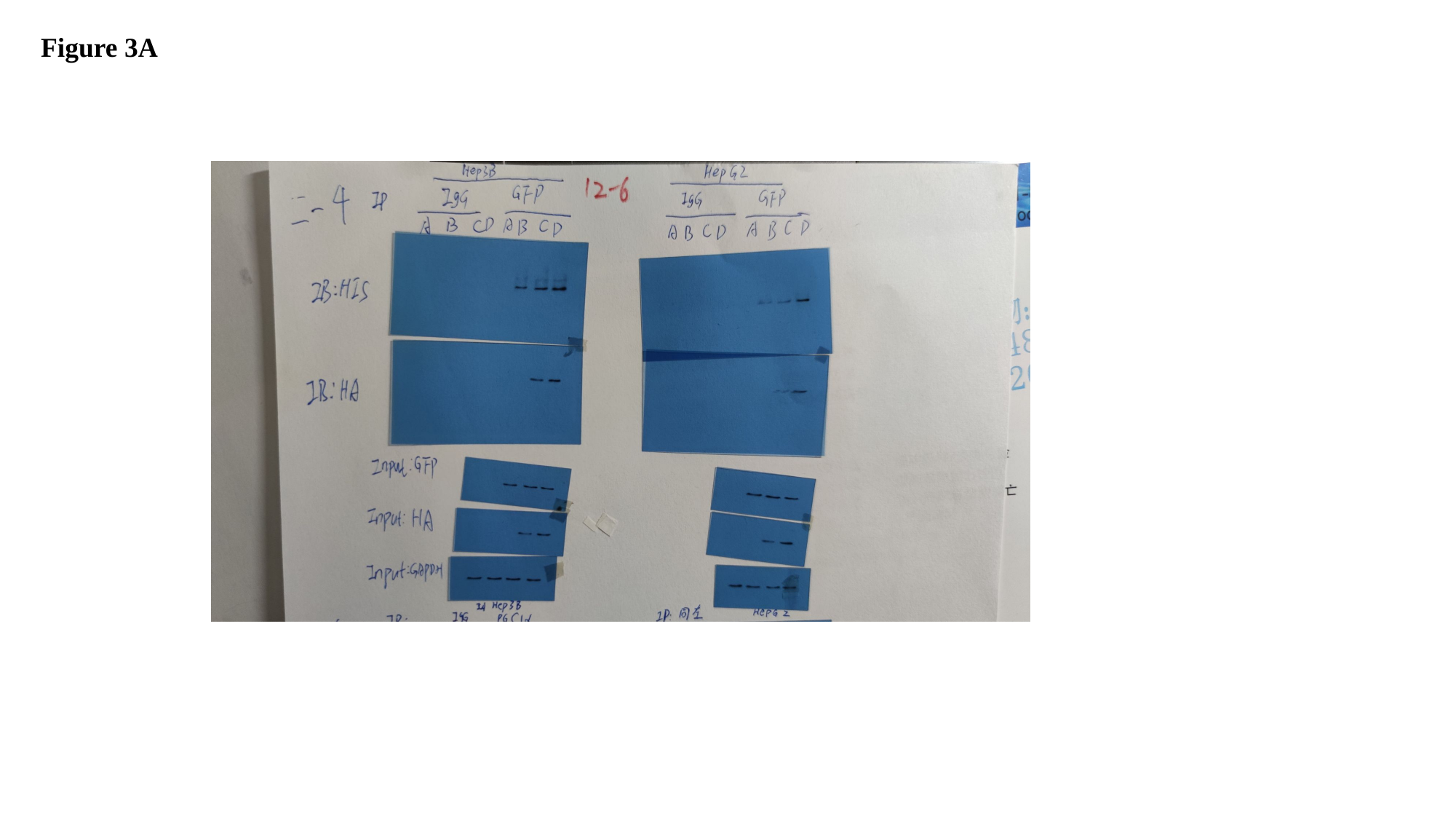

Figure 3A

## Slide 6
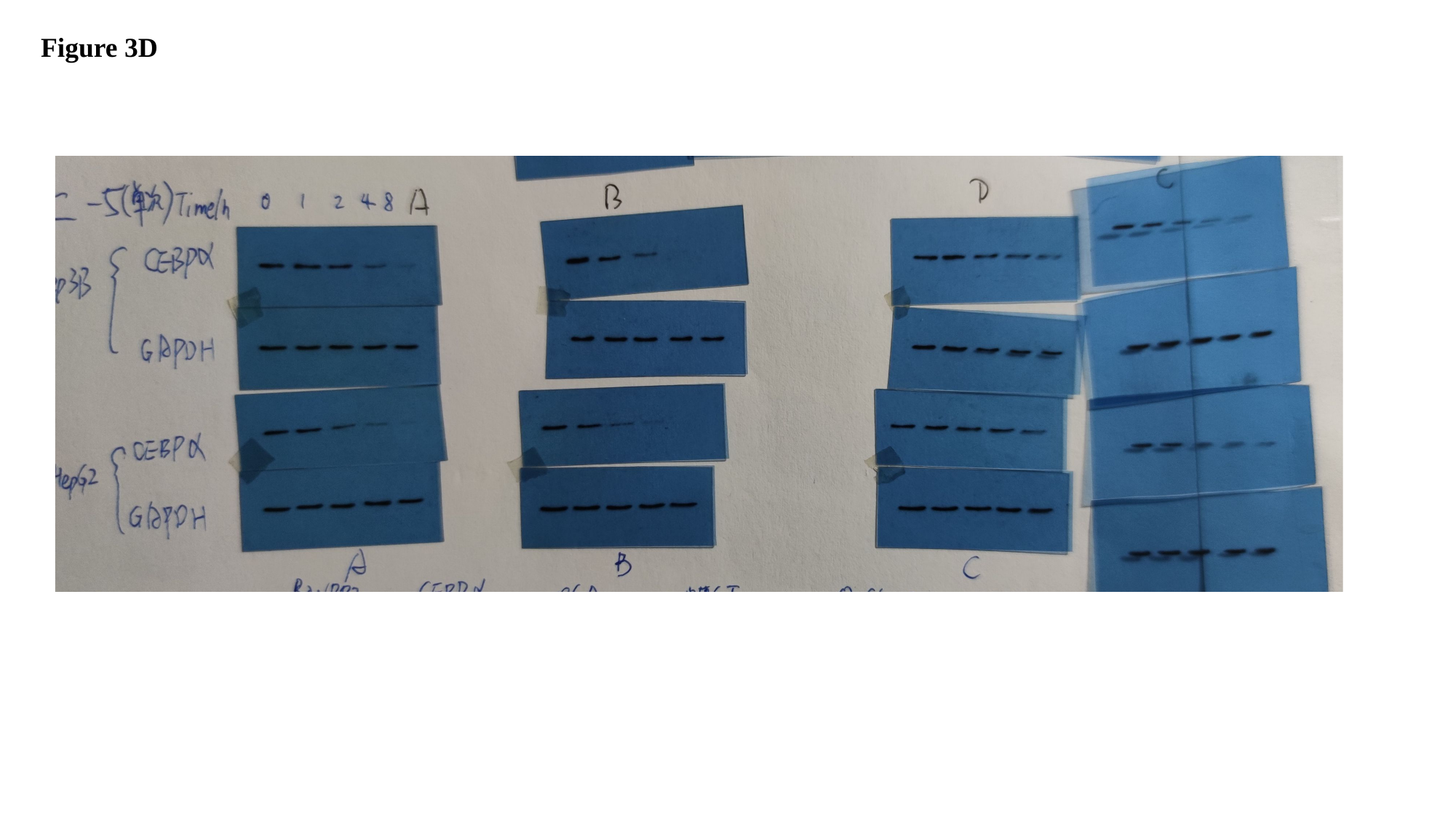

Figure 3D

## Slide 7
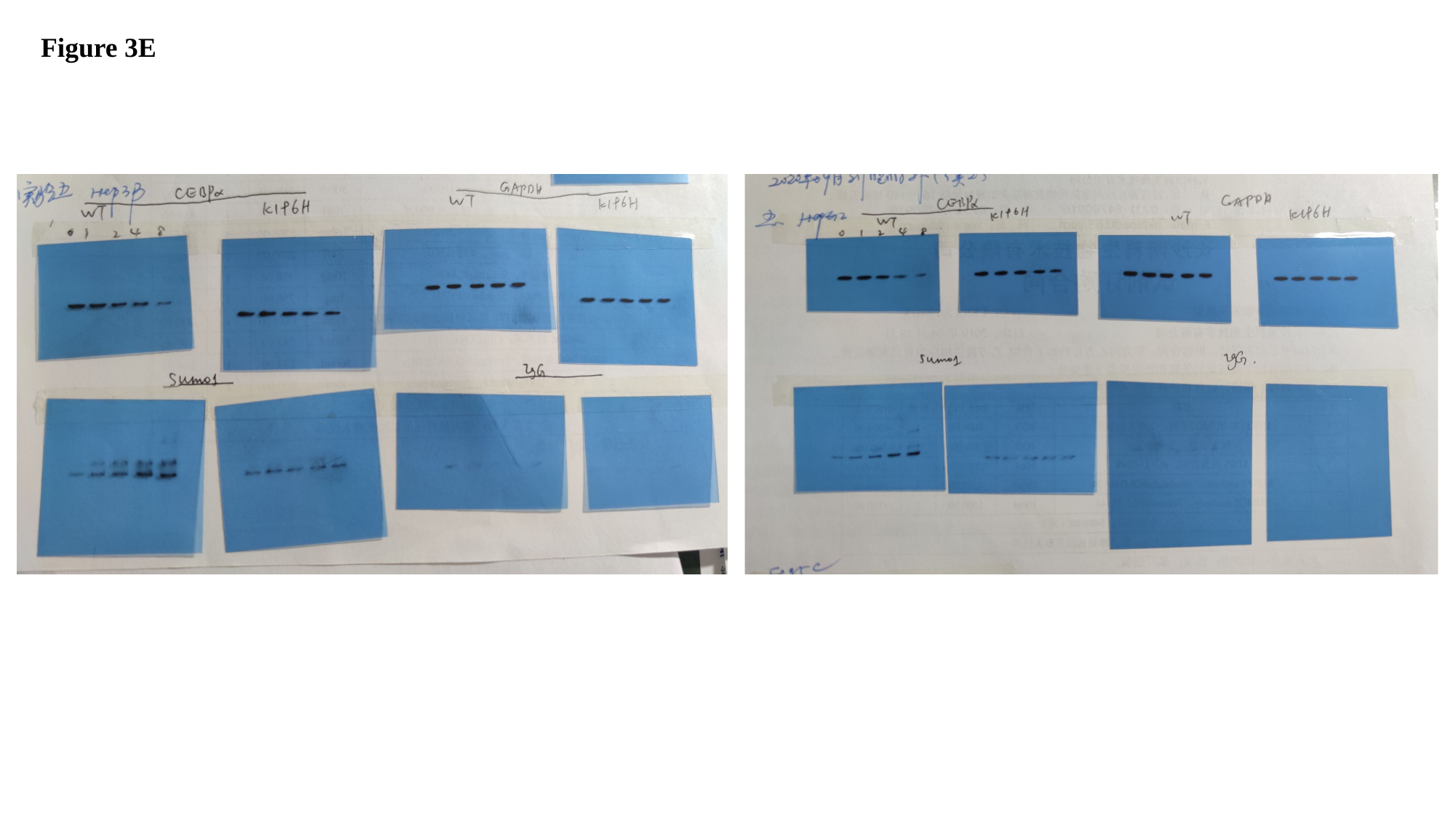

Figure 3E

## Slide 8
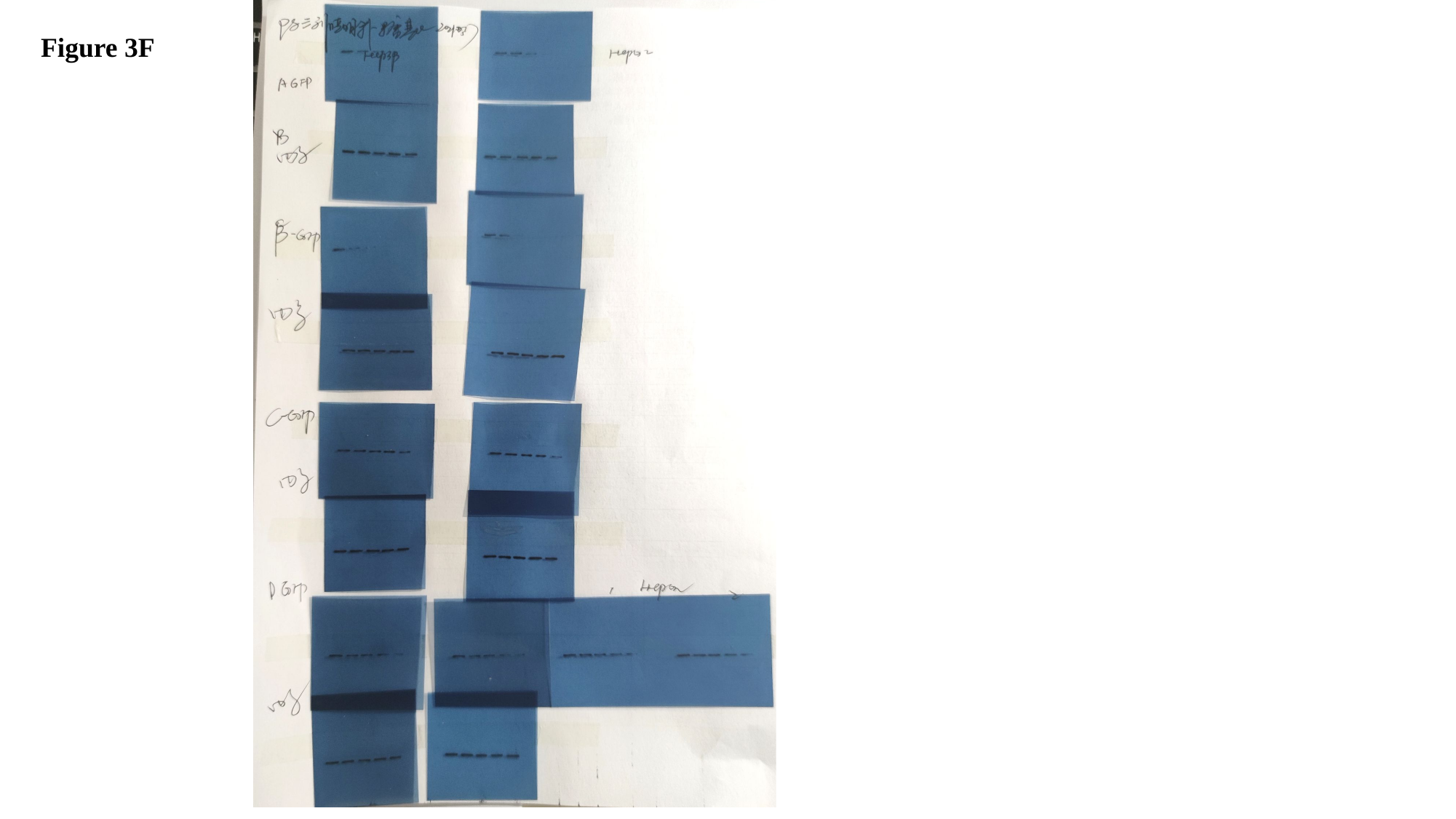

Figure 3F

## Slide 9
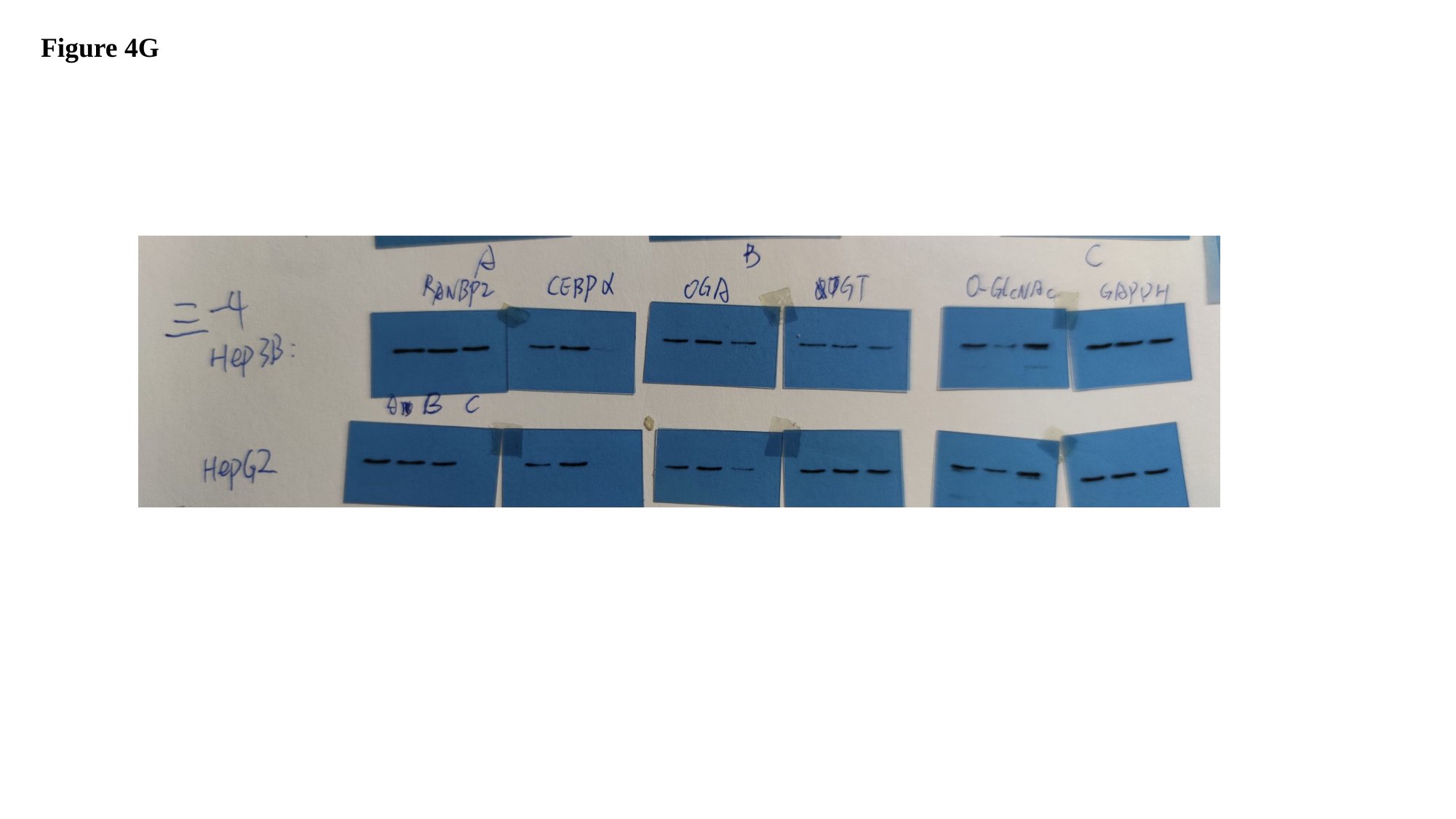

Figure 4G

## Slide 10
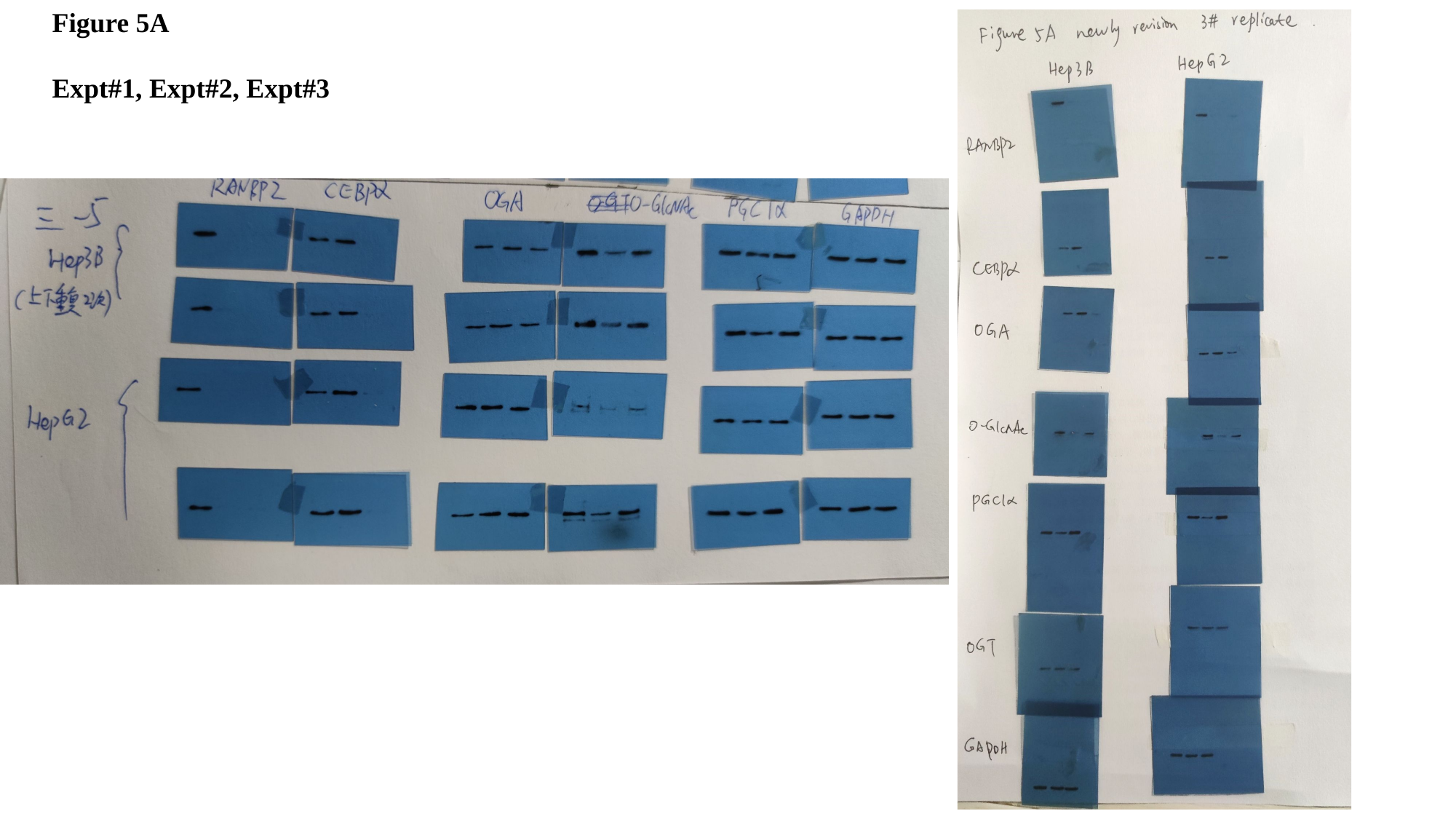

Figure 5A
Expt#1, Expt#2, Expt#3

## Slide 11
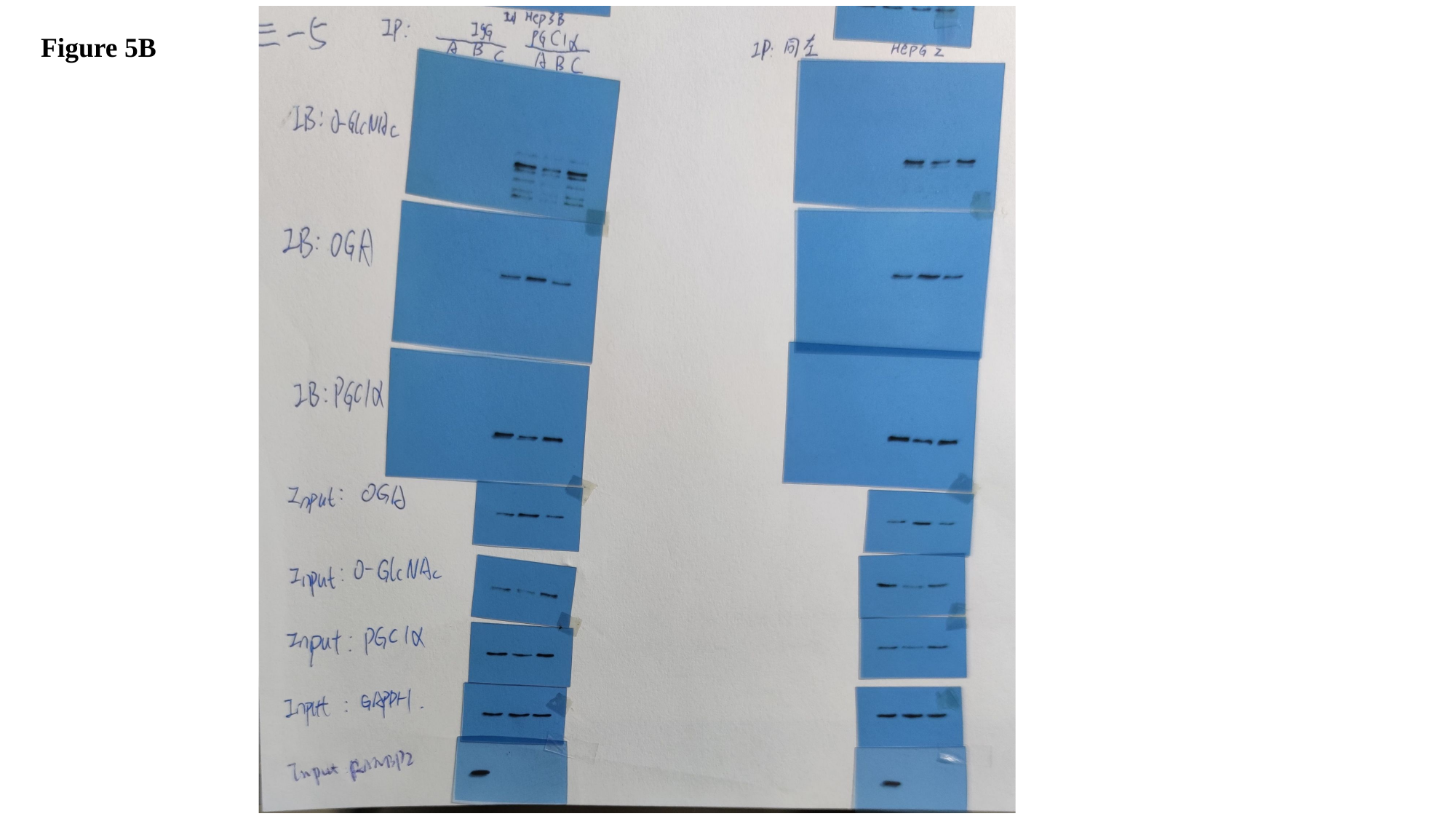

Figure 5B

## Slide 12
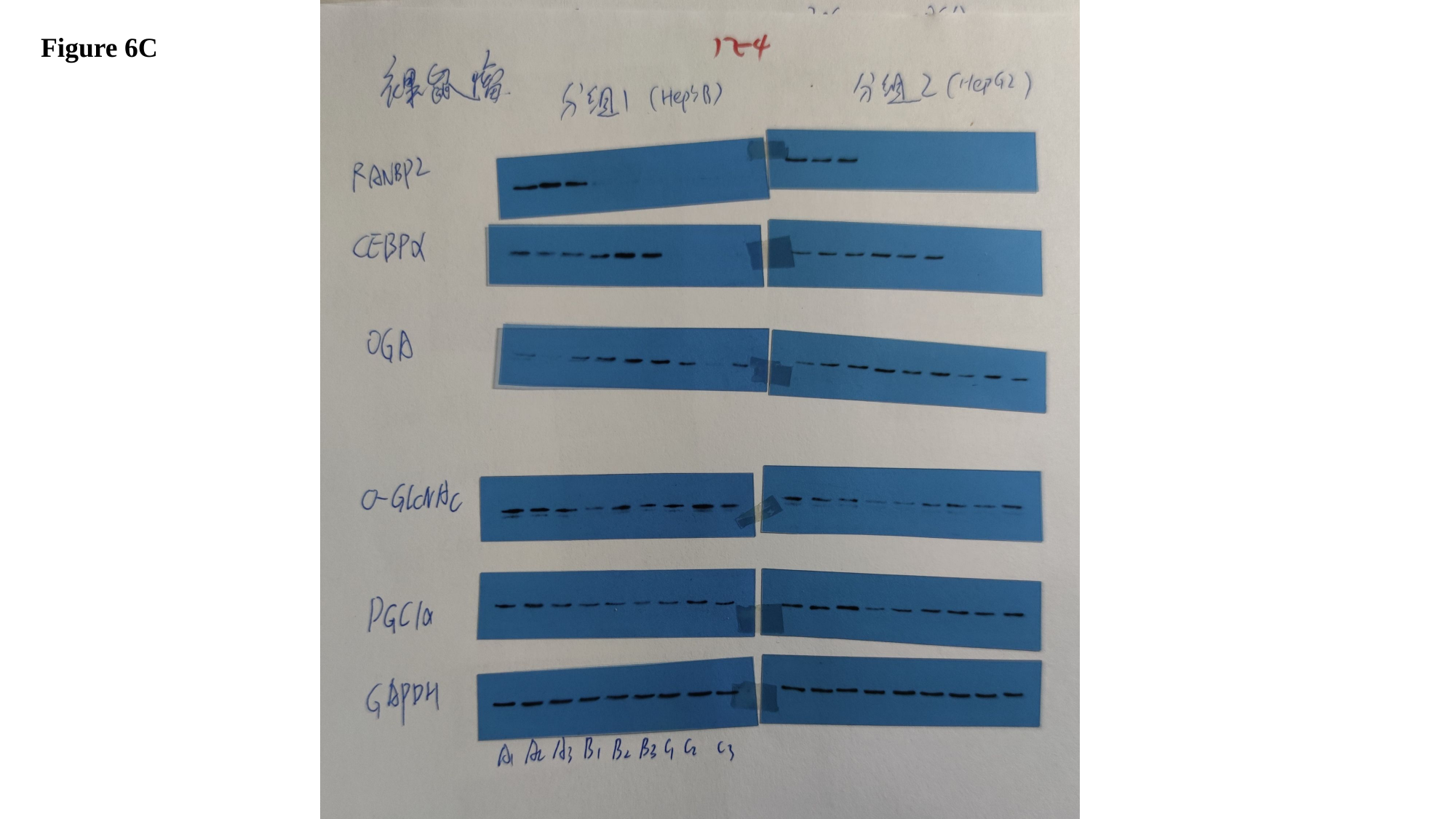

Figure 6C

## Slide 13
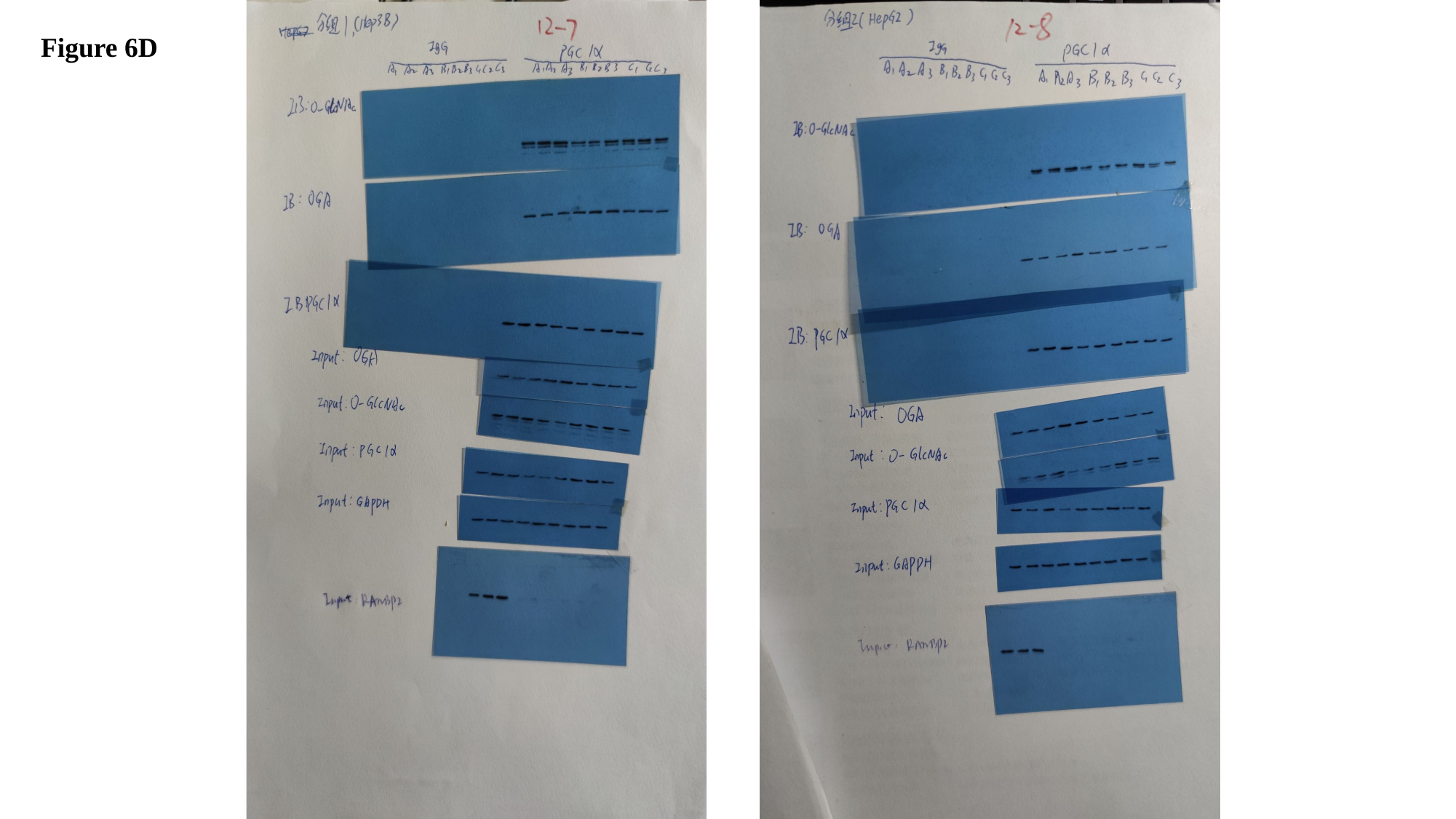

Figure 6D

## Slide 14
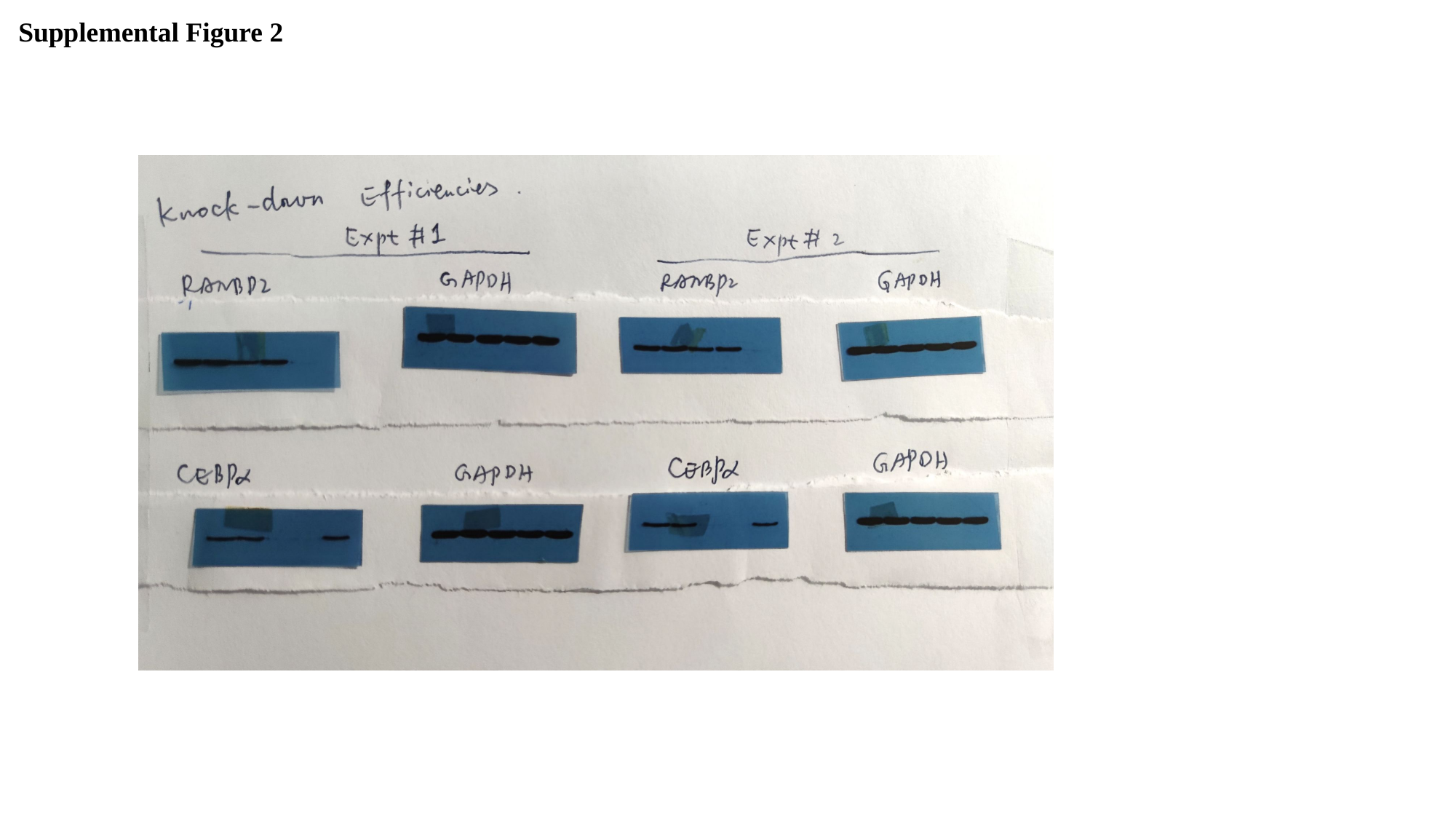

Supplemental Figure 2

## Slide 15
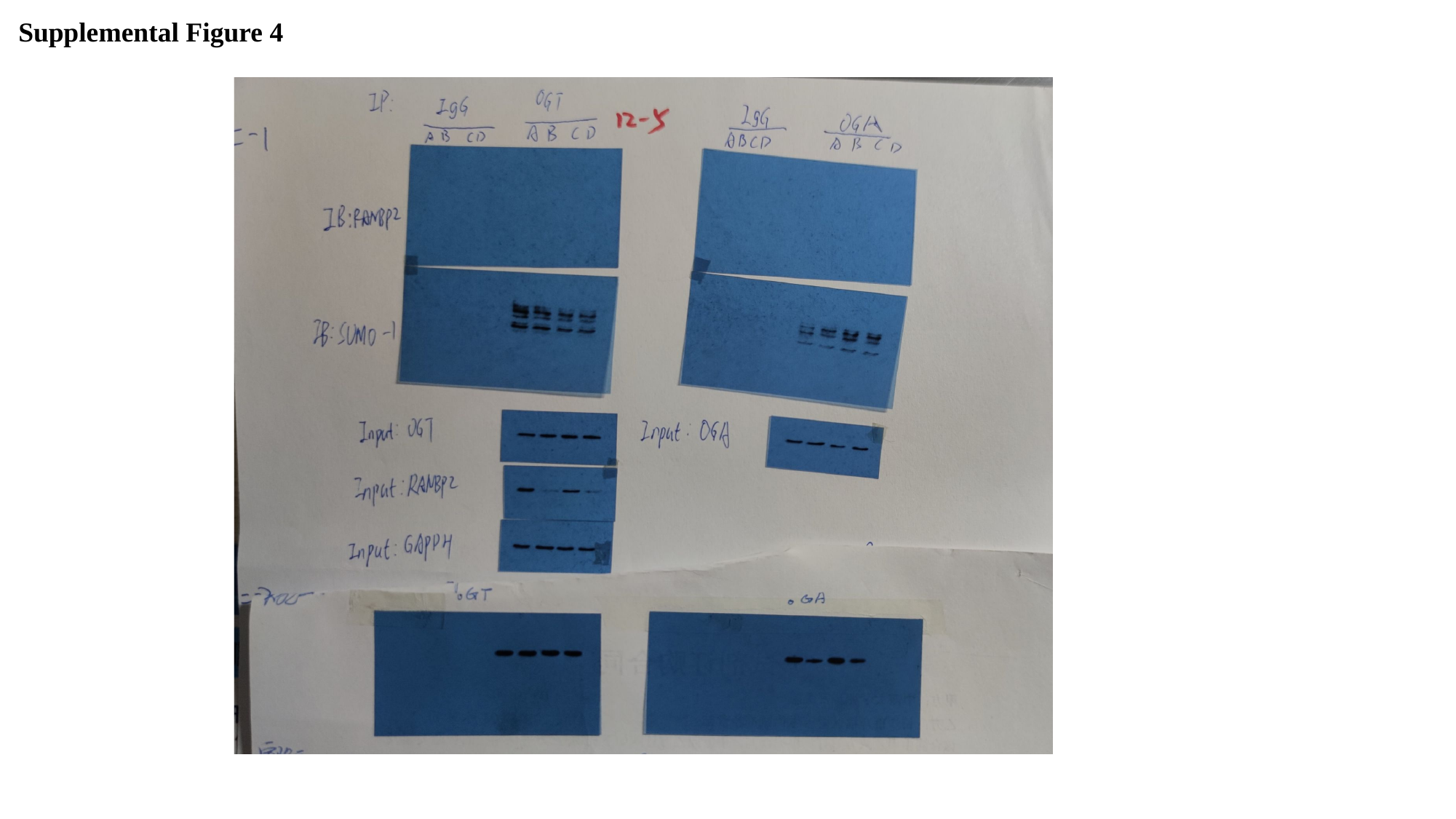

Supplemental Figure 4
